# Structure of POPC Lipid Bilayers in OPLS3e Force Field

**DOI:** 10.1021/acs.jcim.2c00395

**Published:** 2022-08-31

**Authors:** Milla Kurki, Antti Poso, Piia Bartos, Markus S. Miettinen

**Affiliations:** † School of Pharmacy, University of Eastern Finland, Kuopio Campus, Yliopistonranta 1 C, P.O. Box 1627, 70211 Kuopio, Finland; ¶ Department of Chemistry, University of Bergen, 5007 Bergen, Norway; # Computational Biology Unit, Department of Informatics, University of Bergen, 5007 Bergen, Norway

## Abstract

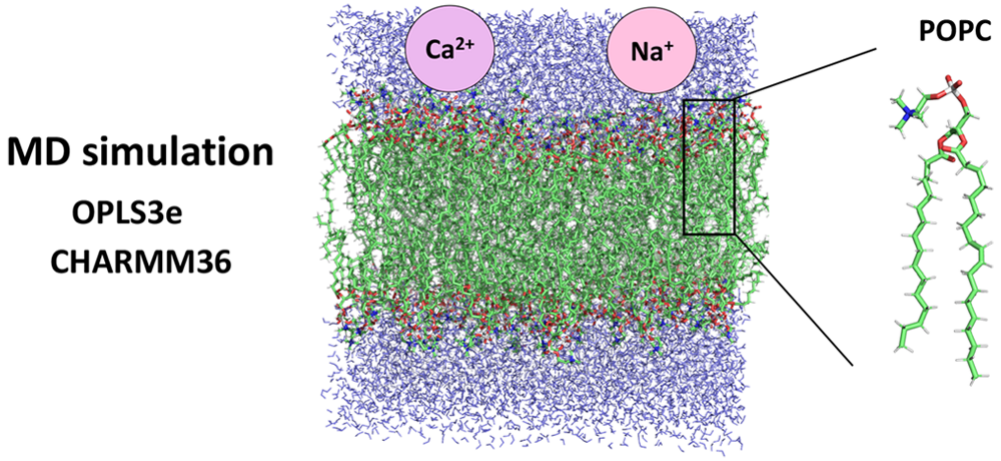

It is crucial for molecular dynamics simulations of biomembranes that the force field parameters give a realistic model of the membrane behavior. In this study, we examined the OPLS3e force field for the carbon–hydrogen order parameters *S*_CH_ of POPC (1-palmitoyl-2-oleoylphosphatidylcholine) lipid bilayers at varying hydration conditions and ion concentrations. The results show that OPLS3e behaves similarly to the CHARMM36 force field and relatively accurately follows the experimentally measured *S*_CH_ for the lipid headgroup, the glycerol backbone, and the acyl tails. Thus, OPLS3e is a good choice for POPC bilayer simulations under many biologically relevant conditions. The exception are systems with an abundancy of ions, as similarly to most other force fields OPLS3e strongly overestimates the membrane-binding of cations, especially Ca^2+^. This leads to undesirable positive charge of the membrane surface and drastically lowers the concentration of Ca^2+^ in the surrounding solvent, which might cause issues in systems sensitive to correct charge distribution profiles across the membrane.

## Introduction

**Figure 1:**
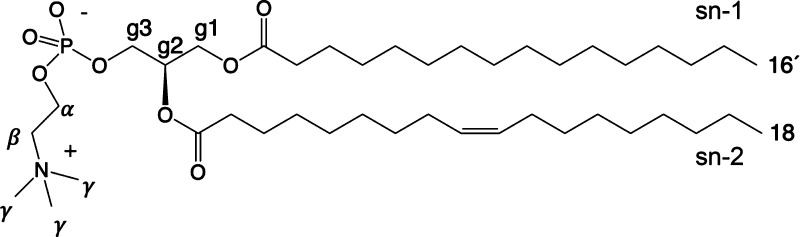
Chemical structure of 1-palmitoyl-2-oleoylphosphatidylcholine (POPC).

Membranes function as biological barriers that separate cells from the environment and delineate different cellular compartments; they are crucial in maintaining the life-sustaining chemical and electrical gradients. The key structural constituents of membranes are phospholipids that form the membrane surface with their polar head groups and the membrane core with their lipophilic tails (Figure 1). In addition to phospholipids, biological membranes contain for example cholesterol, proteins, ions, and oligosaccharides. Lipid bilayers play central role in several biological and pathological processes such as cell division, intracellular membrane trafficking, and formation of lipid rafts.^1,2^ To fully understand these processes, atomistic and molecular level understanding of lipids is required.^3^ Such understanding can be obtained through computational tools, but it is important that those tools depict the structure, dynamics, and function of lipid bilayers accurately. Accurate lipid models allow the reliable study of, for example, membrane-bound proteins, transport through membranes, and pharmacokinetics of drugs.^4−6^

### Why Accurate Force Field Parameters Are Important

Force field accuracy is of key importance in atomistic molecular dynamics (MD) simulation methods. Atomistic MD enables studying systems of interest in life sciences by a balance between computing power and precision: instead of the computationally heavy methods needed to describe quantum mechanical behavior, MD uses typically classical mechanics approximation (called force field) to reproduce molecular behavior. Thus, the validity of MD simulation studies relies heavily on the accuracy of the force field. Not surprisingly, a lot of effort has gone into force field development since the start of MD simulation studies in the 1980s.^7−11^

### OPLS3e Force Field

OPLS3e is one of the most recent updates in the OPLS force field series available in the Schrödinger software suite.^12^ OPLS3e has become widely used in drug discovery and material sciences due to its wide coverage of small molecules and accurate description of protein–ligand interactions. OPLS3e relies heavily on the earlier OPLS3 force field,^13^ but with an addition and refinement of torsional parameters and a better handling of partial charges to offer improved accuracy on small-molecule conformational propensities, solvation, and protein–ligand binding. OPLS3e supports membrane simulations and offers optimized parameters for certain lipids: POPC (Figure 1), DMPC (1,2-dimyristoylphosphatidylcholine), DPPC (1,2-dipalmitoylphosphatidylcholine), and POPE (1-palmitoyl-2-oleoyl-phosphatidylethanolamine). Schrödinger has not, however, made publicly available the OPLS3e force field parameters or the procedure of lipid parameter validation; to our knowledge the accuracy of lipid simulations using OPLS3e, or its predecessor OPLS3, has also not been reported by others. To examine the performance of OPLS3e and to get an insight into how realistic model of lipid bilayers it produces, we simulated pure POPC bilayers at different conditions using the OPLS3e force field. We chose POPC among the OPLS3e-parametrized lipids due to its abundance in biological membranes and the availability of experimental data in the literature.

Performance of a force field can be assessed by comparing different observables between relevant experiments and MD simulations. For membrane lipids, the C–H bond order parameters *S*_CH_ offer an appealing option for such a task since they can be accurately measured experimentally using ^2^H NMR^14^ or ^1^H–^13^C NMR^15−17^ techniques, and easily and directly calculated in MD simulations. The *S*_CH_ have a long history in force field validation for lipids and a large amount of experimental data are available in the literature.^3^ Finally, as *S*_CH_ can be calculated for every C–H bond of the lipid molecule, they offer a very localized picture of the possible deficiencies of the simulation model.^3^

### Lipids in Other Force Fields

Previous studies comparing experimental data to simulations show that in general, acyl chains of lipids are usually rather well described in simulations, and agreement of the structure and behavior of this region between the simulation and the experimental data is quite good.^3,18−20^ However, correct description of headgroups and glycerol backbone has proven to be more challenging, and large variation in performance with different force fields occurs.^21−24^ Predictive power of MD simulations on lipid structure usually decreases close to the water–lipid interfacial region, and more attention for the modeling of this region has been put in lately.^3,25−27^

Atomistic MD simulations of membrane systems have been previously used to research the effects of changing different physiologically relevant conditions, such as the hydration level and ion concentrations.^21,28−32^ Lower hydration is relevant in studying many biological processes, such as membrane fusion;^33^ ions are present in all biological systems, and ion–membrane interactions are of key importance, e.g., in neuron studies.^34−36^ Experimental studies have shown that the phosphatidylcholine headgroup order parameters rise in response to lowering hydration and drop in response to cation binding.^3^ A good-quality atomistic-level force field should also capture these changes.

Response to lowering hydration level is qualitatively correctly produced by several current force fields; but large variation occurs in description of cation binding, which is typically highly overestimated.^21,28,32,37,38^ There are challenges in the correct description of Na^+^ binding, but especially in the correct description of multivalent ions: Ca^2+^ overaccumulates at the membrane–water interface in most of the currently used force fields.^23,24,28^

CHARMM36 is one of the most used lipid force fields; and as it performs quite well in most lipid studies, we use it here as a reference.

In this study, we examined the performance of OPLS3e force field in membrane simulations. We demonstrate that OPLS3e produces C–H bond order parameters for POPC that are very close to experimental values and very similar when compared to the CHARMM36 force field. That said, in OPLS3e, as in many other force fields, the characterization of (especially of Ca^2+^) ion binding to membrane seems problematic.

## Methods

### Order Parameters

In this work the C–H bond order parameters *S*_CH_ are used to assess the force field performance. The *S*_CH_ depend on the angle θ between a C–H bond vector and the membrane normal (in our simulations the *z*-axis direction) as
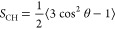
1where the angular brackets denote average over the sampled conformations. Order parameters from simulations can be calculated directly from the atomic coordinates using the eq 1.

Experimental order parameters can be determined for lipid C–H bonds with NMR techniques, such as ^2^H NMR^14^ and ^1^H–^13^C NMR,^15−17^ using quadrupolar splitting and dipolar splitting, respectively. These methods are very accurate and highly sensitive to changes in the lipid structural ensemble.^3^ There are large amounts of experimental *S*_CH_ data available in the literature for different lipids measured with both ^2^H and ^13^C NMR, all in good agreement with each other.^21^ Experimental order parameters have been estimated to have at least ±0.02 accuracy;^15,21^ the error range of 0.02 is used also in this study, as suggested by Botan et al.,^21^ as a sweet spot within which simulated order parameters should ideally reside compared to experimental data. Error range of 0.02 applies to magnitudes but relative changes in *S*_CH_ can be measured with much higher accuracy if the same equipment is used, allowing tracing of minor changes, such as the response to lowering hydration or additional salt;^21,28^ this is utilized also in this study, for more discussion see ref (39).

### Simulations

To compare the OPLS3e and CHARMM36 force fields to experimental data, we performed equally long MD simulations (lengths ranging from 500 ns to 1 μs) using both force fields with matching hydration levels (Table 1) and salt concentrations (Table 2). Here we note just the key simulation details; all details are available in the run input files of the corresponding trajectories on Zenodo, see Tables 1 and 2 for the permanent links. Simulations used standard setup for planar bilayers, zero tension, and periodic boundary conditions in either Desmond, implemented in Schrödinger suite package version 2019.4^40,41^ (OPLS3e) or in GROMACS version 2019.5^42^ (CHARMM36). All simulated systems contained 200 POPC lipids (100 per leaflet) and they were generated using the system builder and model system regeneration tools of the Schrödinger software suite and the CHARMM-GUI Membrane builder, respectively. The NPT ensemble with temperature of 300 K and 1 atm pressure was used. In the simulations containing ions, ions were initially placed randomly in the water phase. Only the steady-state part of the simulations was analyzed, that is, after the bilayer area per lipid (*A*_L_) stabilized and (in salt-containing systems) no further ions accumulated in the membrane. Plots of area per lipid as a function of time are available in Figure S13. Notably, the OPLS3e with high CaCl_2_ concentration did not reach a steady state during 1 μs, so the simulation was divided into 10 × 100 ns parts analyzed separately. Details of the simulations with varying hydration levels are shown in Table 1 and with varying concentrations of additional salt in Table 2.

**Table 1:** Simulated Lipid Bilayer Systems with Varying Hydration Levels^a^

force field	lipid	*N*_w/l_	*N*_w_	*t*_sim_ (ns)	*t*_anal_ (ns)	files
OPLS3e^12^	POPC	44	8859	500	500	(43)
	POPC	20	4000	500	490	(44)
	POPC	10	2000	500	495	(45)
	POPC	5	1000	1000	600	(46)
CHARMM36^47^	POPC	44	8880	500	460	(48)
	POPC	20	4000	500	400	(49)
	POPC	10	2000	500	425	(50)
	POPC	5	1000	1000	650	(51)

a *N*_w/l_: Water/lipid ratio. *N*_w_: Number of water molecules. *t*_sim_: Total simulation time. *t*_anal_: Time used for analysis. Files: Reference containing the simulation files.

**Table 2:** Simulated Lipid Bilayer Systems with Varying Concentration of Additional Salt^a^

force field for lipids/ions	salt	[salt] mM	*N*_w_	*N*_c_	*t*_sim_ (ns)	*t*_anal_ (ns)	files
OPLS3e^12^	NaCl	100	8880	16	1000	1000	(52, 53)
	NaCl	200	8880	32	1000	1000	(54, 55)
	NaCl	500	8880	80	1000	900	(56, 57)
	NaCl	1000	8880	160	1000	900	(58, 59)
	CaCl_2_	50	8880	8	1000	500	(60, 61)
	CaCl_2_	100	8880	16	1000	250	(62, 63)
	CaCl_2_	200	8880	32	1000	10 × 100	(64, 65)
	CaCl_2_	500	8880	80	1000	10 × 100	(66, 67)
	CaCl_2_	1000	8880	160	1000	10 × 100	(68), (69)
CHARMM36/NBFIX^47^^70^	NaCl	100	8880	16	500	475	(71)
	NaCl	200	8880	32	500	455	(72)
	NaCl	500	8880	80	500	440	(73)
	NaCl	1000	8880	160	500	485	(74)
	CaCl_2_	50	8880	8	1000	850	(75)
	CaCl_2_	100	8880	16	1000	850	(76)
	CaCl_2_	200	8880	32	1000	750	(77)
	CaCl_2_	500	8880	80	1000	850	(78)
	CaCl_2_	1000	8726	158	1000	800	(79)

a *N*_w_: Number of water molecules. *N*_c_: Number of cations. *t*_sim_: Total simulation time. *t*_anal_: Time used for analysis. Files: Reference containing simulation files. Salt concentrations are calculated as [salt] = *N*_c_×[water]/*N*_w_, where [water] is 55.5 M.

### Starting Structures and Simulation Details

#### OPLS3e

Starting structures were constructed using the system builder and model system regeneration tools implemented in the Schrödinger software package.^40^ The SPC water model^80^ was used to solvate the systems. In addition, TIP3P^81^ was used as a comparison in a few systems to ensure that the water model does not significantly influence the order parameters (see SI section 1 for details). For the dehydrated systems, excess water was removed from the starting structure of the full hydration system to attain the different hydration states. For the ion-containing systems, numbers of ions were calculated as *N*_c_ = [salt] × *N*_w_/[water], where [water] = 55.5 M. The system with the strongest ion concentration (1 M) was constructed first using the system builder, and other concentrations were generated by randomly removing excess ions. Simulations were performed using Desmond in Schrödinger suite’s package version 2019–4.^40,41^ Default settings for membrane systems were used, with 2 fs time step and saving data every 10 ps; systems were relaxed before simulations with the default membrane relaxation protocol of Desmond. Temperature was set at 300 K and the system was kept in the NPT ensemble with the semi-isotropic Martyna–Tobias–Klein barostat^82^ and the Nosé–Hoover-chain thermostat.^83^

#### CHARMM36

The starting structures were constructed using the CHARMM-GUI Membrane Builder (www.charmm-gui.org).^84^ CHARMM TIP3P water model^85,86^ was used to solvate the systems. Different hydration states were generated by removing excess water from the systems. Ions were added by using the gmx genion tool with -np and -nn flags in the GROMACS software package.^42^ All simulations were performed with GROMACS version 2019.5.^42^ Force field parameters were taken as in the CHARMM-GUI outputs; consequently, the NBFIX parameters^70^ were used for ions. Simulations were performed with a 2 fs time step and data saved every 10 ps. Temperature of 300 K was maintained with the Nosé–Hoover thermostat,^87,88^ and the semi-isotropic Parrinello–Rahman barostat^89^ was used to control the pressure.

#### Analysis

The C–H bond order parameters *S*_CH_ were calculated directly using the eq 1. The *S*_CH_ of each C–H bond was gained by calculating first the *S*_CH_ of each individual lipid over time separately, and then calculating the average and the standard error of mean over different lipids. This analysis was performed using the Python program calcOrderParameters.py from NMRlipids GitHub;^90^ the program uses the MDAnalysis library.^91,92^ Number densities were obtained by using the gmx density tool in GROMACS software package.^42^ Desmond files were converted for analysis into GROMACS format using VMD^93^ for trajectories and convert.py by Intermol^94^ for other files. After the convert.py conversion, names of the waters and ions, and representation of ions as individuals (instead of box of ions) were manually modified to match the other files.

The area per lipid *A*_L_ for each simulation frame was determined by calculating the area of the simulation box in the *xy*-plane and dividing that by the number of lipids per leaflet (*n* = 100); for the average *A*_L_, only the equilibrated part of simulations (*t*_anal_) was used.

The small-angle X-ray scattering (SAXS) form factors were calculated using the Python program form_factor.py in NMRlipids GitHub^95^ and normalized by the first peak (located between 0.1 and 0.2 1/Å) height.

Lipid lateral diffusion coefficients *D*_L_ were determined from the slope of the mean squared displacement (MSD) of lipid centers of the mass. First the Gromacs tool gmx trj (with the -nojump and -com flags) was used to create trajectories of lipid centers of mass (for the equilibrated part of simulations *t*_anal_, see Tables 1 and 2); then the tool gmx msd (with the -lateral z and -rmcomm flags) was used to calculate the MSD. The MSD was then plotted as a function of the displacement time, its linear region determined for each simulation, and *D*_L_ obtained as 0.25 × the slope (of a line fit to the linear region). For error estimation (visualized in Figure S14), the lipids were divided into five subgroups of equal size (*n* = 40), and MSDs calculated for each subgroup; the resulting five MSDs were treated as independent measurements, allowing the standard error of mean to be calculated for each displacement time; the steepest and gentlest slopes of lines that fit within these standard errors of the mean then provided error estimates for *D*_L_.

## Results and Discussion

We calculated the C–H bond order parameters *S*_CH_, see eq 1, from the simulations performed at different conditions, see Tables 1 and 2, with the OPLS3e and CHARMM36 force fields and compared them to the experimental *S*_CH_ available in the literature.

### Validation against NMR Order Parameters: Full Hydration

**Figure 2:**
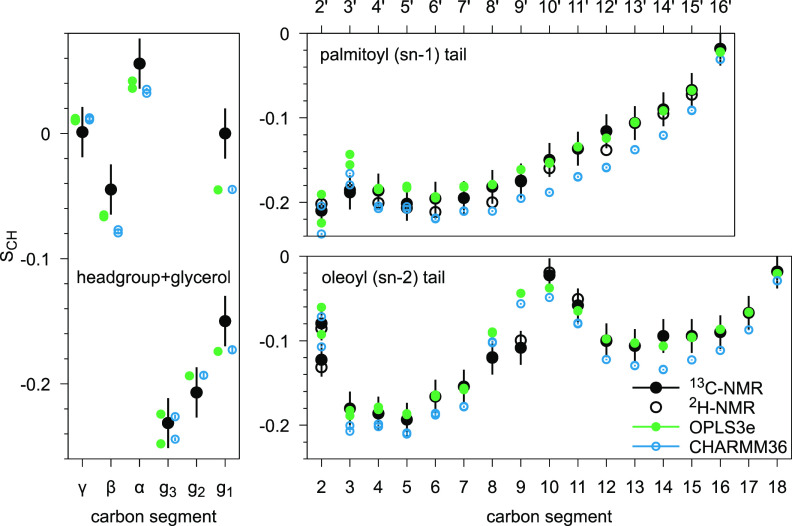
Carbon–hydrogen bond order parameters *S*_CH_ at full hydration for headgroup, backbone, and acyl chains in simulations and experiments. Experimental values for the POPC ^1^H–^13^C NMR at 300 K are from ref (17) and for ^2^H NMR from ref (96). The ±0.02 error bars of the ^13^C experimental values represent also the range within which most of the published experimental data resides, see discussion in text. For naming of carbon segments, see Figure 1.

Most *S*_CH_ produced by OPLS3e reside within ±0.02 from the experimental values, that is, within the estimated error range of NMR experiments (Figure 2). However, problems with *S*_CH_ magnitude occur in *g*_1_, near the double bond of the sn-2 chain (C9), and at the start of sn-1 chain. For these regions the experimental error range is not reached with either of the force fields. Whereas OPLS3e and CHARMM36 produce almost identical *S*_CH_ for headgroup and glycerol backbone, the performance of OPLS3e for acyl chain regions seems to surpass CHARMM36.

**Figure 3:**
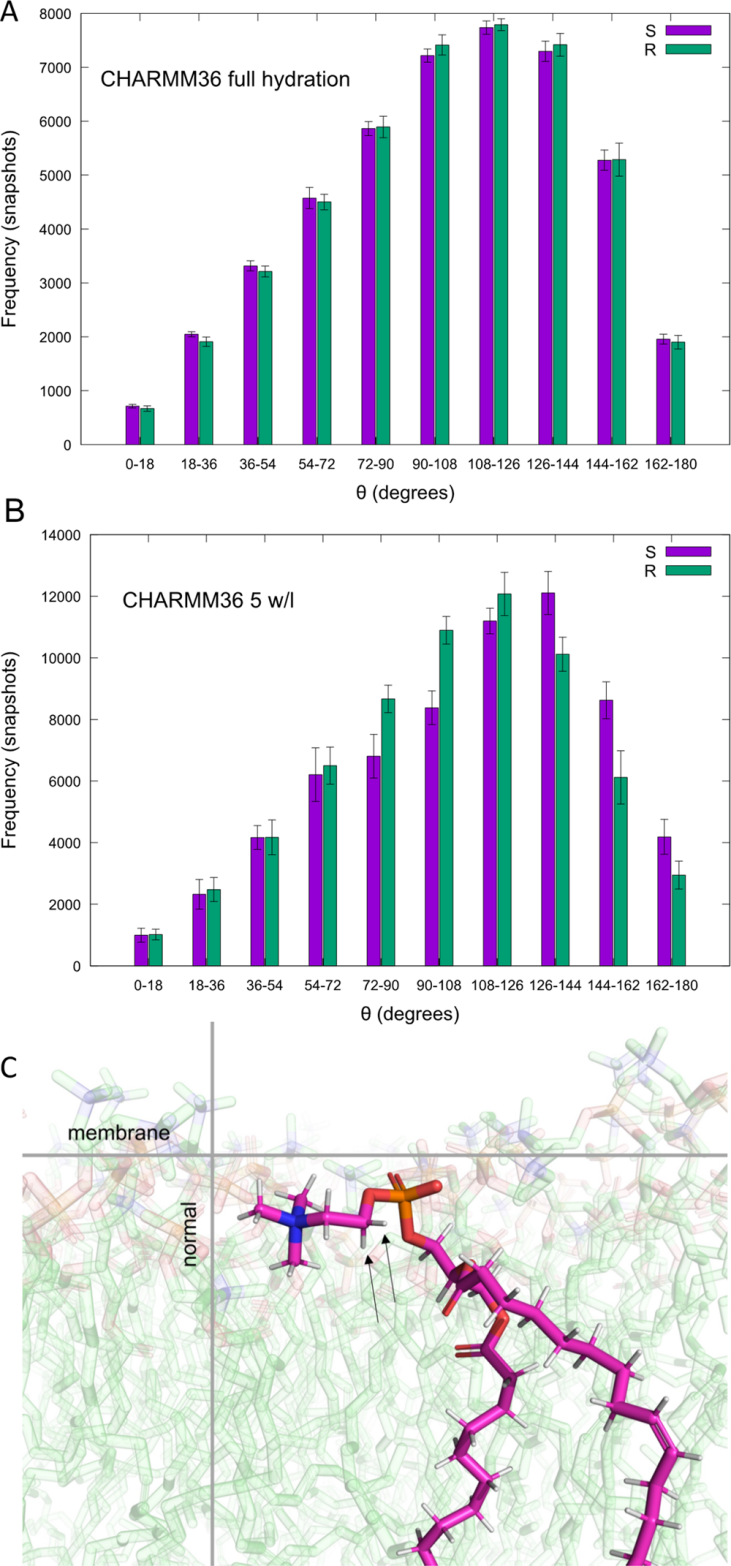
Illustration of forking. The θ-angle distribution of the α-carbon C–H bonds for R and S hydrogens (A) in CHARMM36 at full hydration (44 w/l) showing no forking and (B) in CHARMM36 at 5 w/l showing forking, cf. Figure 4. Distributions are calculated over 5 lipids as an example; error bars represent standard error of mean. θ is the angle between a C–H bond and the membrane normal; see Methods for more information. (C) A snapshot with the α-carbon hydrogens marked with arrows.

In addition to magnitudes, a high-fidelity simulation model should produce correct forking pattern of *S*_CH_. The term forking is used to describe the occurrence of unequal *S*_CH_ for different hydrogens attached to the same carbon, indicating different orientational populations of the two C–H bonds. It has been shown to not result from two separate populations of lipids.^97,98^ Based on experimental data, most carbons of POPC have equally sampled C–H bond orientations and produce equal *S*_CH_ for both hydrogens; but there are few exceptions: the R and S hydrogens attached to the *g*_1_ and *g*_3_ carbons^17,97^ in the glycerol backbone show in experiments significant and moderate forking, respectively, and the C2 carbon of sn-2 chain shows moderate forking, see Figure 2. An accurate force field should produce correct forking for *g*_1_, *g*_3_, and C2 but show no forking for other carbons. Forking is illustrated in the Figure 3 by angle distributions toward membrane normal. In CHARMM36 at full hydration, angle distributions for both hydrogens attached to the α carbon are equal (Figure 3A), but at 5 w/l (Figure 3B) distributions are unequal showing forking.

At full hydration, OPLS3e and CHARMM36 correctly produce forking for *g*_1_ and *g*_3_ and C2 of sn-2 chain. The C2 of sn-2 chain is of particular interest, as several force fields have been shown to struggle in this area.^3,22^ However, both force fields produce forking for C2 and C3 at the start of sn-1 chain, which is not in agreement with the experimental data.

In general, OPLS3e produces very similar pattern of order parameters at full hydration as CHARMM36: close to experimental values but not within experimental accuracy. However, both force fields have problems with the correct description of g_1_ at glycerol backbone, the beginning of the acyl tails, and the double bond of oleoyl chain. Therefore, we can conclude that OPLS3e produces comparable *S*_CH_ to CHARMM36 at full hydration, suggesting that structural description of POPC is similar and reasonably accurate in these force fields.

### Validation against NMR Order Parameters: Dehydration

**Figure 4:**
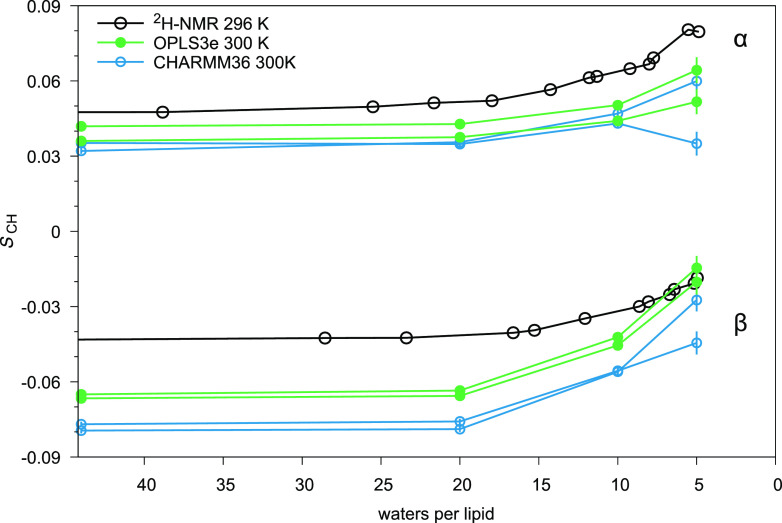
Response of the headgroup order parameters *S*_CH_^β^ and *S*_CH_^α^ to decreasing hydration level. Experimental values for POPC (^2^H NMR) at 296 K are from ref (99). Notably, small changes in temperature seem not to have a major effect on *S*_CH_, see Figures S10 and S11.

To examine how decreasing hydration affects the performance of OPLS3e, we compared the headgroup order parameters *S*_CH_^β^ and *S*_CH_^α^ from OPLS3e and CHARMM36 simulations against experimental NMR data (Figure 4). In experiments, *S*_CH_^β^ and *S*_CH_^α^ rise when hydration level drops, a change that should be captured in simulations. Although the *S*_CH_^β^ are not within ±0.02 from experimental data (Figure 4), which indicates that neither force field exactly produces the atomistic resolution structural ensemble of the headgroup, the changes produced in *S*_CH_^β^ and *S*_CH_^α^ are qualitatively in line with experimental data: Both increase as hydration level decreases in OPLS3e, and in CHARMM36. Also, the magnitude of the rise for *S*_CH_^α^ in OPLS3e aligns with the experimentally measured rise, but for *S*_CH_^β^ the rise is exaggerated. Similar observations can be made with CHARMM36; however, an additional forking not reported in previous studies is occurring in CHARMM36 at the low hydration level of 5 w/l, see Figures 3 and 4. Our simulation length at 5 w/l (1000 ns, see Table 1) was reasonably long compared to earlier simulation studies reporting *S*_CH_^β^ and *S*_CH_^α^ at low hydration,^21^ resulting in small error estimates and making the difference between the C–H bonds clearly visible.

Based on these data, structural response to dehydration seems quite realistic for the headgroup in OPLS3e. Botan et al. suggest an intuitive explanation for the rising headgroup order parameters to be that the choline headgroup orients more parallel to the membrane as the interlamellar space shrinks in response to the decreasing hydration level.^21^ To conclude, in response to dehydration, OPLS3e does not produce atomistic resolution but its performance is very similar to CHARMM36 and headgroup orientation in OPLS3e can be thought to be reasonably accurate under dehydrated conditions. Although many currently available force fields produce qualitatively correct response to lowering hydration, OPLS3e and CHARMM36 seem to be among the most realistic considering the magnitude of the response.

### Validation against NMR Order Parameters: Ions

Order parameters of the phosphatidylcholine (PC) headgroup C–H bonds can be used to compare ion binding to lipid membranes in experiments and simulations.^28^ Charged objects on a PC bilayer interface induce systematic changes for the order parameters of the α and β carbons: A positive charge induces a decrease, and a negative charge an increase in *S*_CH_^β^ and *S*_CH_^α^; a lack of change in *S*_CH_^β^ and *S*_CH_^α^ implies that the charged object does not bind to the PC bilayer interface. The concept, often referred to as the “molecular electrometer”, has a strong experimental background^100^ and Catte et al. have demonstrated that also in atomistic MD simulations, the *S*_CH_^β^ and *S*_CH_^α^ act as a direct indicator for bound cation charge.^28^ Therefore, comparison of ion affinities between experiments and simulations based on the PC headgroup order parameters is possible, and allows the assessment of simulation model quality at different salt concentrations.

**Figure 5:**
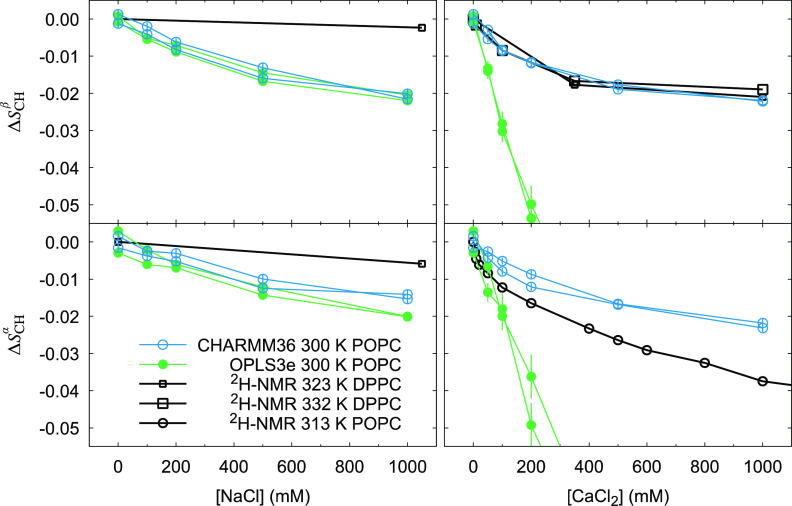
Change of order parameters in the headgroup α (lower panels) and β (upper panels) segments in response to rising concentrations of NaCl (left panels) or CaCl_2_ (right panels). Experimental values for DPPC (^2^H NMR) at 323 and 332 K are from ref (101) and for POPC (^2^H NMR) at 313 K from ref (102). The out-of-bounds Δ*S*_CH_^β^ points of OPLS3e in response to CaCl_2_ (top right panel) are −0.102 ± 0.0085 and −0.089 ± 0.0090 (500 mM), and −0.13 ± 0.011 and −0.11 ± 0.013 (1000 mM). Corresponding values for Δ*S*_CH_^α^ (bottom right panel) are −0.10 ± 0.010 and −0.093 ± 0.010 (500 mM), and −0.073 ± 0.016 and −0.097 ± 0.015 (1000 mM). Full figure is shown as the Figure S3. Due to their very slow equilibration (see Figure S4), for the OPLS3e CaCl_2_ 200, 500, and 1000 mM concentrations the last 100 ns of the 1 μs simulation was used here. Note that to show possible forking at [salt] = 0, best seen in the bottom left panel for the OPLS3e, the average of the C–H bond order parameters of the R and S hydrogens was used to set the baseline.

**Figure 6:**
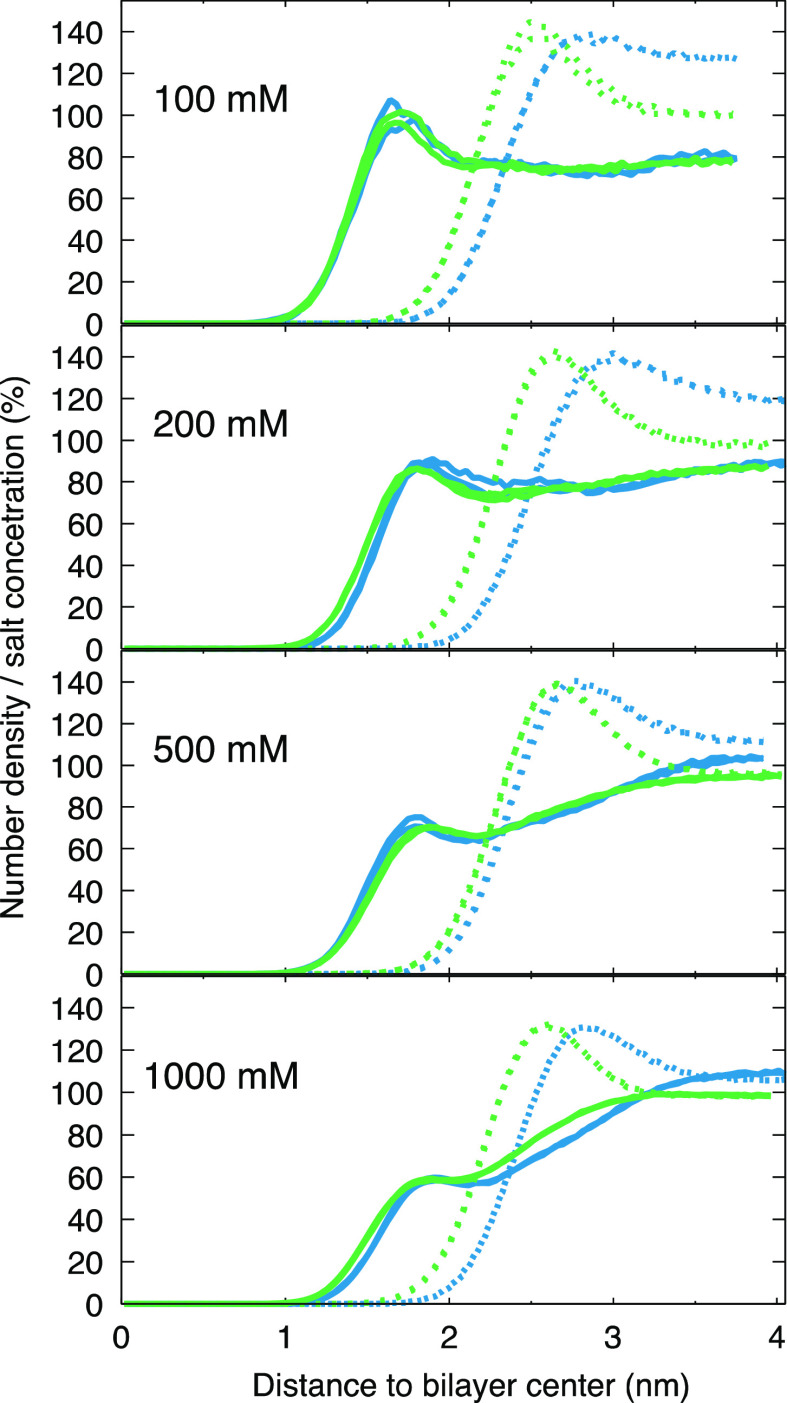
Distribution for Na^+^ (solid lines) and Cl^–^ (dashed) ions along the bilayer normal shown as percentage of salt concentration. Green represents OPLS3e and blue CHARMM36. The graphs were obtained by dividing the number densities with the total salt concentration. Note that both leaflets are plotted (two almost fully overlapping lines) to highlight the symmetry of the ion distributions.

In the experiments, adding NaCl induces minimal decrease to *S*^α^_CH_ and *S*^β^_CH,_ suggesting minimal Na^+^ binding to membrane.^101^ Several MD force fields overestimate Na^+^ binding, and consequently *S*_CH_^α^ and *S*_CH_^β^ drop significantly more than in the experiments.^28^ Based on the headgroup order parameter change (Figure 5 left panels) and ion distributions (Figure 6) in our simulations, OPLS3e is not an exception: Na^+^ binding is overestimated. Notable is that overestimation is visible also at lower concentrations, near the physiological 150 mM concentration, that have the highest relevance in life sciences. Shapes of the order parameter curves in response to rising concentration of NaCl are very similar in OPLS3e and CHARMM36 (Figure 5 left panels). Also, distributions of Na^+^ and Cl^–^ seem highly similar (Figure 6), which suggests similar response to rising concentrations of NaCl in both force fields. For CHARMM36, we have included the nonbonded fix (NBFIX) corrections for ion parameters that have been suggested to recover overestimation of Na^+^ binding;^103^ however, the NBFIX-corrected CHARMM36 still appears to overestimate Na^+^ binding (Figure 5. left panels).

Distributions of Na^+^ and Cl^–^ (Figure 6) show that at lower salt concentrations (100 and 200 mM) ions are not only accumulated in the vicinity of the membrane, but they are also unevenly distributed in the bulk solution (Na^+^ and Cl^–^ curves do not converge in the bulk); meaning that even with the rather large water/lipid ratios used here, the desired effect of having equal concentrations of both ions in the bulk solution is not reached in either of the force fields with [NaCl] < 500 mM.

**Figure 7:**
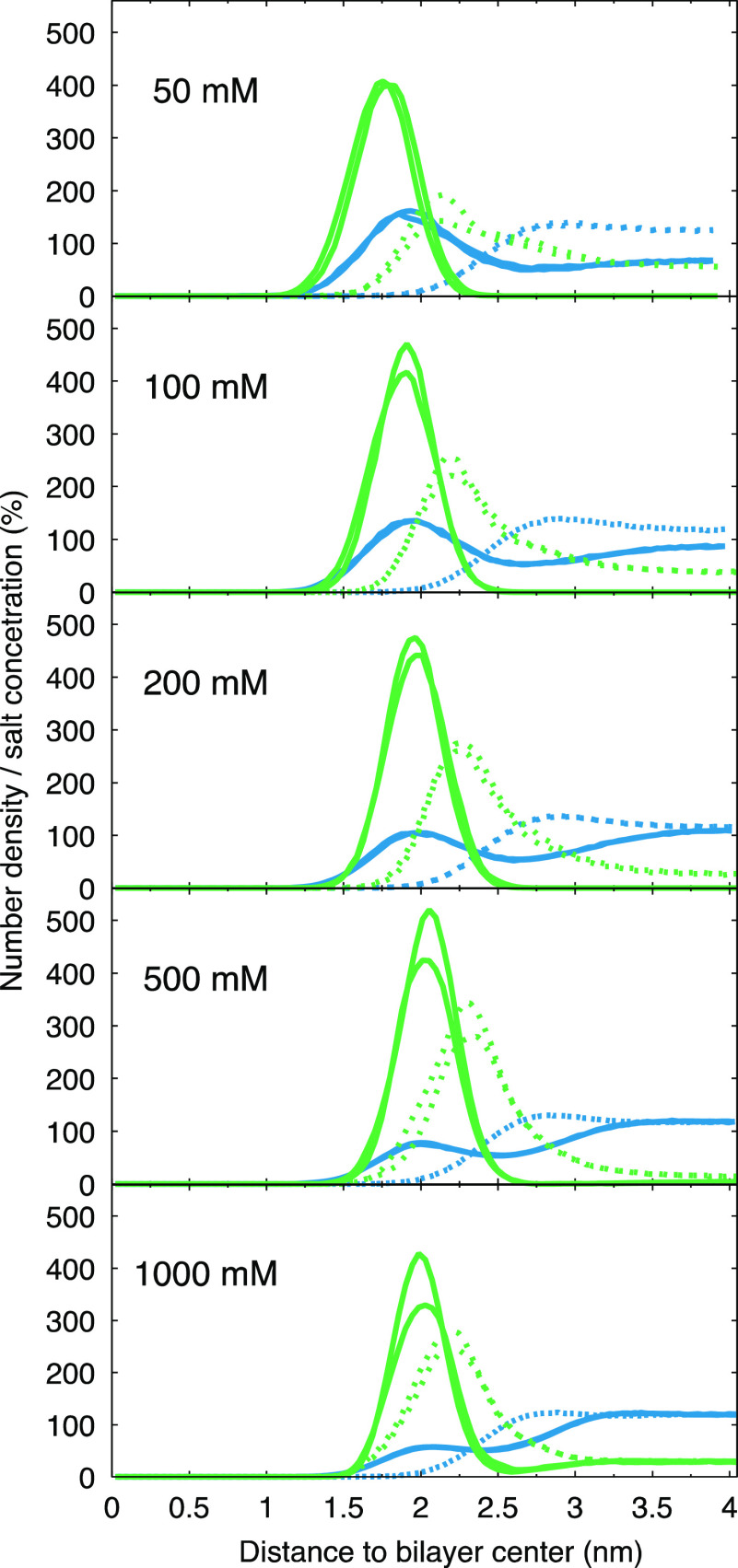
Distribution for Ca^2+^ (solid lines) and Cl^–^ (dashed lines) ions along the bilayer normal shown as percentage of salt concentration. Green represents OPLS3e and blue CHARMM36. The graphs were obtained by dividing the number densities with the total salt concentration. Because of their very slow equilibration (see Figure S4), for the OPLS3e CaCl_2_ 200, 500, and 1000 mM concentrations the last 100 ns of the 1 μs simulation was used here. Note that both leaflets are plotted (two mostly overlapping lines).

Contrary to Na^+^ ions, divalent Ca^2+^ ions bind significantly to lipid bilayers in experiments and the PC headgroup order parameters decrease when CaCl_2_ concentration increases. Correct description of calcium binding to bilayers has proven to be challenging, and it seems that of the current force fields only the ECC-POPC model produces a quantitatively accurate response.^23,28,32^ However, many force fields can have qualitatively right response to Ca^2+^, but overestimate the binding affinity. OPLS3e too produces qualitatively right order parameter response to Ca^2+^ ions: *S*_CH_^β^ and *S*_CH_^α^ decrease with rising concentration of Ca^2+^; but the decrease is far too great (Figure 5 right panels) and binding of Ca^2+^ is highly overestimated (Figure 7). Order parameters of CHARMM36 are closer to experimental data than OPLS3e, suggesting that OPLS3e produces a poorer response to additional CaCl_2_ than CHARMM36. However, *S*_CH_^α^ of CHARMM36 suggest a slight underestimation of Ca^2+^ binding, and the NaCl and CaCl_2_ responses seem very much alike, suggesting that—as already previously indicated in the SI of ref (23)—CHARMM36 with NBFIX parameters does not distinguish the difference between monovalent Na^+^ and divalent Ca^2+^ seen in the experiments.

Overestimation of ion binding in OPLS3e can be seen also in ion distributions, especially for CaCl_2_ (Figure 7). Membrane pulls all calcium ions from the solution at low (≤200 mM) concentrations (and nearly all at higher concentrations), leaving no Ca^2+^ ions to the bulk. A high density of Ca^2+^ ions can be seen at the membrane surface, and consequently, the neutral PC bilayer will appear as positively charged, pulling a high density of Cl^–^ ions next to the Ca^2+^. This charge layering will result in a strong electrostatic gradient. Such an artificially charged membrane can distort MD simulation results; in addition to effects on membrane behavior, influence on charged domains of membrane proteins is also conceivable, and might underlie some contradicting MD simulation results.^104,105^ Therefore, extreme caution should be exercised when simulating membrane protein systems containing ions using force fields that are known to overestimate cation binding.

It is worth keeping in mind that as calcium binding of OPLS3e is so highly overestimated that (nearly) all Ca^2+^ is bound to bilayer, and no Ca^2+^ is left to solution, studying effects of calcium solutions on systems containing membranes using OPLS3e will be very difficult.

To conclude, additional NaCl produces similar response in both OPLS3e and CHARMM36: Slight overestimation of sodium binding compared to the minimal binding suggested by experimental data. On the contrary, responses to CaCl_2_ differ in our MD simulation between OPLS3e and CHARMM36. OPLS3e produces qualitatively right response to rising CaCl_2_ concentration, but radically overestimates the Ca^2+^ binding. Response of CHARMM36 seems to be closer to the experiments, although it is not able to reproduce the difference between Na^+^ and Ca^2+^ ions. The commonly occurring overestimation of cation binding poses one of the biggest problems in current membrane modeling using MD simulations as it may result in positively charged membrane which can qualitatively distort the results.

Several strategies to fix the overbinding of ions to membranes have been proposed in the literature. NBFIX, which was used for CHARMM36 in this study, addresses the issue by tuning nonbonded parameters for specific atom pairs separately instead of using the standard arithmetic combining rule.^70^ An alternative solution is the electronic continuum correction (ECC) strategy, which takes electronic polarization effects of solvent into account by scaling the charge of the ions.^29,32^ We tested a similar scaling as in the ECC model for NaCl and CaCl_2_ in OPLS3e which, prescaling, overestimates Na^+^ and Ca^2+^ binding to membrane (Figure 5). Scaling of charge by 0.75 for Na^+^ and Cl^–^ ions decreased Na^+^ binding to membrane, and order parameters were closer to experimental values (than without scaling, see Figure S6). For CaCl_2_, a similar simple scaling of ionic charges did not fix the surplus ion binding to the membrane (Figures S7 and S8). In the ECC-POPC model^32^ scaling of both ions and the partial charges of lipid headgroup atoms was conducted, resulting in one of the most realistic force fields for lipids so far, since it can produce accurate binding also for divalent Ca^2+^ which, as already previously stated, has been very challenging with all current force fields.

For both force fields used in this study, OPLS3e and CHARMM36, new releases have been published recently. OPLS4 was published in 2021, with updates, e.g., on the representation of hydration and treatment of molecular ions.^106^ There were, however, no changes in membrane parametrization. New CHARMM36 parameters called as C36/LJ-PME were also published in 2021,^25^ with parameters optimized for lipid membranes using a semiautomated approach and including long-range dispersion via Lennard-Jones particle-mesh Ewald (LJ-PME). Order parameters were used as one of the optimization targets and, based on ref (25), order parameters for headgroup and tails of DPPC and DMPC lipids at full hydration align rather well with the experimental data. It will be interesting to see if these new updates offer improved performance for membranes especially in the presence of ions, which has been challenging area to be simulated correctly by the earlier force fields.

### Applications: Responses of Area Per Lipid to Hydration and Ions

**Figure 8:**
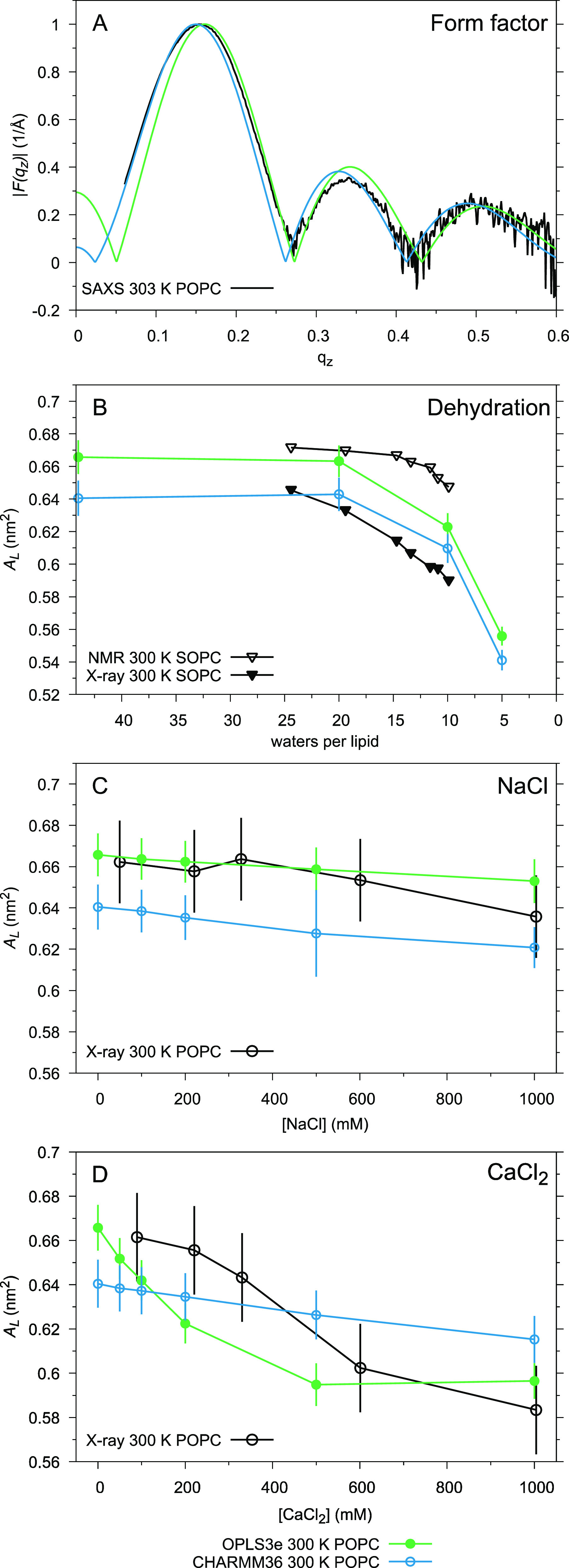
(A) Small-angle X-ray scattering (SAXS) form factors at full hydration (44 w/l); experimental data from ref (107). The responses of area per lipid *A*_L_ to lowering hydration level (B), added NaCl (C), and added CaCl_2_ (D); error bars represent standard deviation. Experimental *A*_L_ as a function of lowering hydration for SOPC (NMR and X-ray) are from ref (110) and as a function of [NaCl] and [CaCl_2_] for POPC from ref (111).

Area per lipid (*A*_L_) is one of the most intuitive descriptors of a lipid bilayer, and thus one of the most common choices when force fields are validated against experimental data.^8,9^ However, *A*_L_ cannot be directly measured experimentally, which complicates the exact comparison of experiments and simulations. For validation purposes, a direct comparison to the (X-ray and neutron) scattering form factors—which reflect the *A*_L_ and other membrane properties such as the bilayer thickness—is, thus, preferable.^3^Figure 8A presents such comparison of our OPLS3e and CHARMM36 simulations at full hydration against the small-angle X-ray scattering (SAXS) form factor of Kučerka et al.;^107^ it is seen that while neither force field perfectly captures the membrane dimensions (e.g., CHARMM36 slightly misses the location of *F*(*q*_*z*_) = 0 between 0.25 and 0.30 1/Å, OPLS3e is somewhat off at short wave vector lengths (*q*_*z*_ < 0.15 1/Å), and both force fields overestimate the relative height of the lobe located between 0.3 and 0.4 1/Å), they do reproduce the experimental form factor quite well. This is in line with the fact that our calculated full hydration *A*_L_ (0.666 ± 0.010 nm^2^ in OPLS3e and 0.640 ± 0.011 nm^2^ in CHARMM36) are well within the range of values reported in the experimental literature for POPC close to our simulation temperature of 300 K: This spans from 0.593 nm^2^ (^2^H NMR, 301 K, ref (108)) to 0.683 nm^2^; (X-ray scattering, 303 K, ref (107)).

Instead of attempting quantitative comparisons of *A*_L_, it is interesting to perform qualitative comparisons against experimentally observed responses in *A*_L_ with changing conditions. Figure 8B shows that *A*_L_ drops similarly in response to lowering hydration in our POPC simulations as what was experimentally^110^ observed for SOPC (1-stearoyl-2-oleoyl-phosphatidylcholine) lipid bilayers (Figure 8B). The experimental data were only available for limited w/l ratios, but the magnitude of the drop in simulations correlates relatively well with experimental data in the available range; this indicates that OPLS3e and CHARMM36 capture rather well not only the PC headgroup behavior (Figure 4) but also the dehydration-associated changes in bilayer dimensions.

With additional NaCl, *A*_L_ do not change significantly at submolar concentrations in either experiments or simulations; both force fields, however, do reproduce the gently descending trend (Figure 8C) despite their overestimation of the headgroup response (Figure 5). With additional CaCl_2_, *A*_L_ shows a significant drop within the experimentally studied concentrations (Figure 8D); CHARMM36 seems to underestimate this, while in OPLS3e the magnitude of the drop is in line with the experimentally determined change. When the latter is, however, viewed in light of the massive overestimation of the headgroup response (Figure 5), resulting from exaggerated affinity for Ca^2+^ (Figure 7), and the below-discussed overestimation of decrease in lateral diffusion, it is highly likely to result from a fortuitous cancellation of errors.

### Applications: Lateral Diffusion

As lipid diffusion along biomembranes occurs on time scales accessible in MD simulations, and the lateral diffusion coefficients *D*_L_ can be determined using several (e.g., fluorescence, electron paramagnetic resonance (EPR), or NMR spectroscopy) experiments, the *D*_L_ are often compared between simulations and experiments. Problematic for some of the experimental techniques (such as EPR and fluorescence methods) is the requirement of labeling probes, which may have an impact on diffusion rates, making interpretation of results more challenging.^9^ As NMR methods do not require system modifications with probes, we here use NMR data as reference.

Our CHARMM36 simulations at full hydration produced *D*_L_ = 5.7 ± 0.3 × 10^–12^ m^2^/s, well in agreement with the values observed in earlier studies^112,113^ at similar (∼20 to ∼50 ns) displacement times; while for OPLS3e diffusion is slightly faster, *D*_L_ = 6.5 ± 0.4 × 10^–12^ m^2^/s, it still appears somewhat slower than in NMR experiments, where *D*_L_ = 8.87 × 10^–12^ m^2^/s at 298 K and 10.7 × 10^–12^ m^2^/s at 303 K.^114^ One must keep in mind, however, that correcting for the finite periodic size of the simulation could increase *D*_L_ by even 200% in CHARMM36.^115^ Indeed, the finite-size correction^116^ using a membrane shear viscosity of 50.7 mPas (Matti Javanainen, personal communication) provides an estimate of 15 × 10^–12^ m^2^/s for CHARMM36. This is considerably faster than what is observed experimentally—an interesting discrepancy in the light that CHARMM36 does reproduce the conformational dynamics of POPC very well.^117^

Nevertheless, as accurate determination of *D*_L_ from simulations is well-known^9^ to be sensitive to various details, such as the simulation box size, measurement length, the observed displacement time, and the ability to apply corrections that in turn depend for example on the accurate determination of viscosity, we refrain from further quantitative study here. Instead, we turn to look at qualitative responses of *D*_L_ to changing conditions; here the observed trends should hold, if the membrane viscosity can be considered constant.

**Figure 9:**
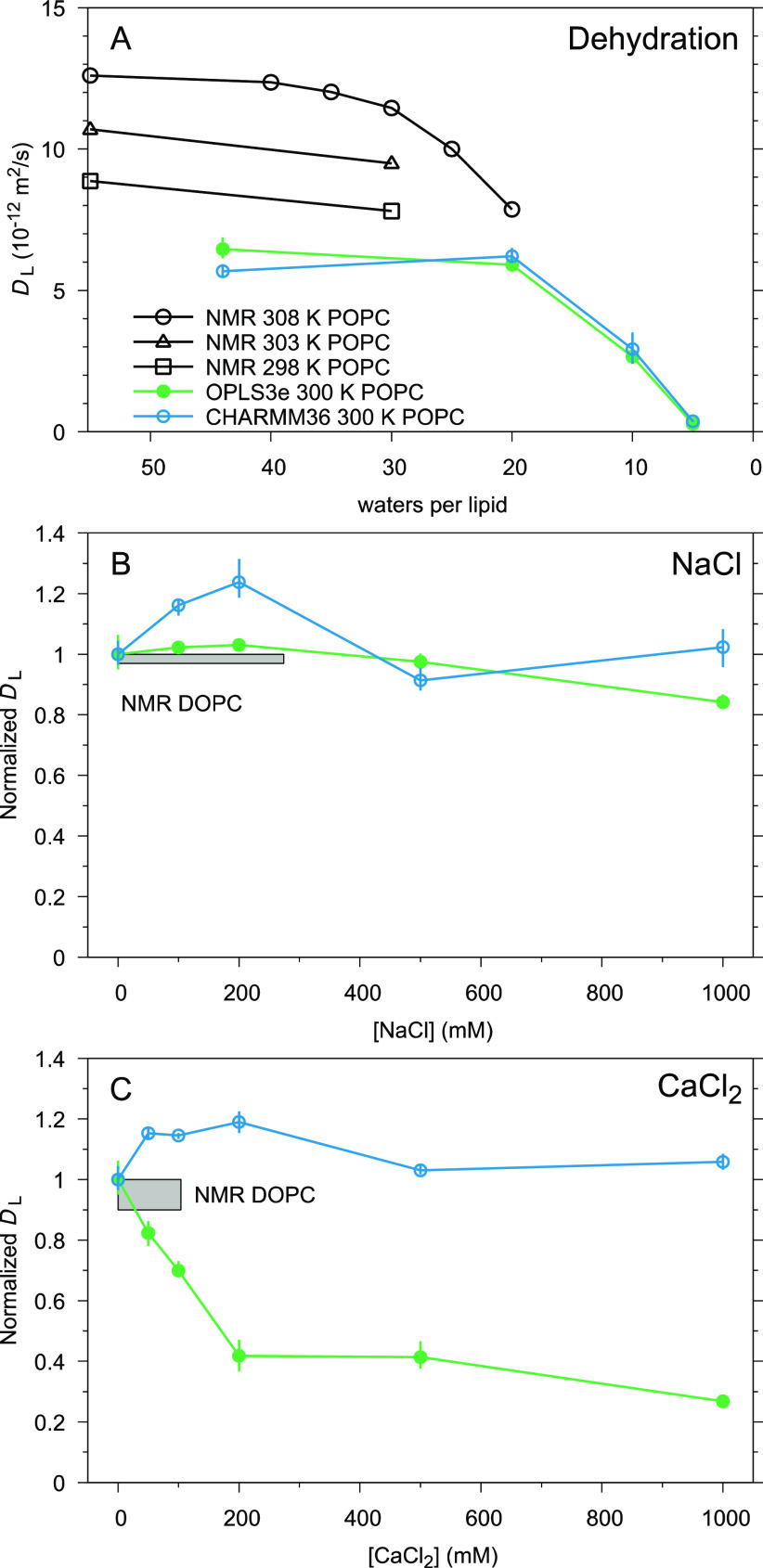
Responses of lipid lateral diffusion coefficients *D*_L_ to lowering hydration level (A), added NaCl (B), and added CaCl_2_ (C). The experimental data in panel A are from ref (114), except for the 30 w/l values for 303 and 298 K, which are from ref (119). Note that panels B and C show *D*_L_ normalized with respect to the salt-free state, as the relevant experimental data in ref (118) were given as maximal percentage changes (indicated here with the gray rectangles).

Figure 9A shows that response of diffusion to lowering hydration is qualitatively correct in both OPLS3e and CHARMM36: *D*_*L*_ drops significantly. However, the response begins later (at lower hydration levels) in simulations (<20 w/l) than in experiments (<40 w/l).

The two bottom panels in Figure 9 show the relative (normalized by the salt-free *D*_L_) response of *D*_L_ to added NaCl (Figure 9B) and CaCl_2_ (Figure 9C). The gray boxes in both panels indicate the reported experimental change: For [NaCl] below 270 mM a decrease of less than 3% was observed and for [CaCl_2_] below 100 mM a decrease of less than 10%;^118^ this suggests that at such concentrations of NaCl or CaCl_2_ do not have major effect on lipid movements. In our simulations, additional NaCl in the experimental range (<270 mM) did not cause a decrease of *D*_L_ in either force field—but rather an increase, especially in CHARMM36 (Figure 9B). A similar increase was observed in CHARMM36 also with added CaCl_2_ (Figure 9C), in line with the finding that the NBFIX parameters result in similar binding behavior for Ca^2+^ as for Na^+^ (see Figures 5–7). In OPLS3e, however, additional CaCl_2_ induced a significant drop of *D*_L_ (Figure 9C), in line with the distortedly strong Ca^2+^ binding (see Figures 5 and 7). This Ca^2+^-induced suppression of movement of neutral POPC lipids in OPLS3e appears, in fact, to be as strong as what Filippov et al. reported (in the same salt concentration range) for the negatively charged DOPG (dioleoylphosphatidylglycerol) lipids, for which they observed an up-to-37% decrease in *D*_L_ upon cation binding.^118^

## Conclusions

MD simulations using accurate force fields allow studying biomembranes at different conditions to interpret experimental results and to get knowledge of membrane structure, as well as the function and dynamics of membrane-bound proteins. Here, we demonstrate the performance of OPLS3e to be reasonably accurate for POPC membranes at full hydration and upon dehydration: The C–H bond order parameters produced with OPLS3e behave similarly to the well-established membrane force field CHARMM36 and closely follow the experimental observations. We also demonstrate that, in the absence of salt, OPLS3e simulation of a fully hydrated POPC bilayer produces the experimentally observed small-angle X-ray scattering form factor as well as an area per lipid that is well within the experimentally reported range. However, extreme caution should be exercised with systems containing ions, especially Ca^2+^ ions: OPLS3e, similarly to most other force fields, overestimates the binding of the cationic ions to the membrane, which both disrupts the neutral net charge of the membrane surface and changes the concentration of ions in the surrounding solvent. These issues could affect, for example, the structure and dynamics of the charged domains of membrane-bound proteins in unexpected ways.

Our results confirm that OPLS3e can be used for reliable MD simulations with simple POPC bilayers. Future studies should elucidate its performance with more diverse bilayers, such as ones containing mixtures of different lipids or cholesterol.

## Supporting Information Available

Additional details of the impact of TIP3P water model in OPLS3e, order parameters in response to additional salt, equilibration times of the ion binding and impact on order parameters, scaling the ion charge in OPLS3e, small bug in CHARMM36 parameters, effect of temperature change in CHARMM36, and area per lipid as a function of time and lipid mean squared displacement (MSD) (PDF)

## Notes

The scripts for the bond order parameter analysis are freely available at NMRlipids GitHub (https://github.com/NMRLipids) and the analyzed trajectories are permanently freely available at Zenodo (https://zenodo.org/).
