# Supplementary material for: Structure of POPC Lipid Bilayers in OPLS3e Force Field
Source: J Chem Inf Model. 2022 Aug 31;62(24):6462–74. doi: 10.1021/acs.jcim.2c00395 (PMC9795559; doi:10.1021/acs.jcim.2c00395)
Supplement: Supplementary file 1 — ci2c00395_si_001.pdf [file ci2c00395_si_001.pdf]

Supporting information:

Structure of POPC lipid bilayers in OPLS3e force field

Milla Kurki[1], Antti Poso[1], Piia Bartos[1]‡, Markus S. Miettinen[2*]‡*

‡These authors contributed equally.

[1]University of Eastern Finland, School of Pharmacy, Kuopio Campus, Yliopistonranta 1 C, P.O. BOX 1627, 70211 Kuopio

[2]Department of Chemistry, University of Bergen, Bergen, Norway

Computational Biology Unit, Department of Informatics, University of Bergen, 5007 Bergen, Norway

*to whom correspondence should be addressed.

*Email: piia.bartos@uef.fi

Content

1. Impact of TIP3P water model in OPLS3e
2. Order parameters in response to additional salt
3. Equilibration times of the ion binding and impact on order parameters
4. Scaling the ion charge in OPLS3e
5. Small bug in CHARMM36 parameters
6. Effect of temperature change in CHARMM36
7. Area per lipid as a function of time
8. Lipid mean squared displacement (MSD)

1. Impact of TIP3P water model in OPLS3e

To clarify if water model has an impact on cation binding affinity of membranes in OPLS3e, we performed simulations using the TIP3P water model instead of SPC with 1000 mM concentration of NaCl or $CaCl_2$, and without ions. Without ions (Fig. S1), and at 1000 mM concentrations (Fig. S2), TIP3P produced similar order parameters as SPC, suggesting that there is no major difference between SPC and TIP3P water models in OPLS3e with or without ions.

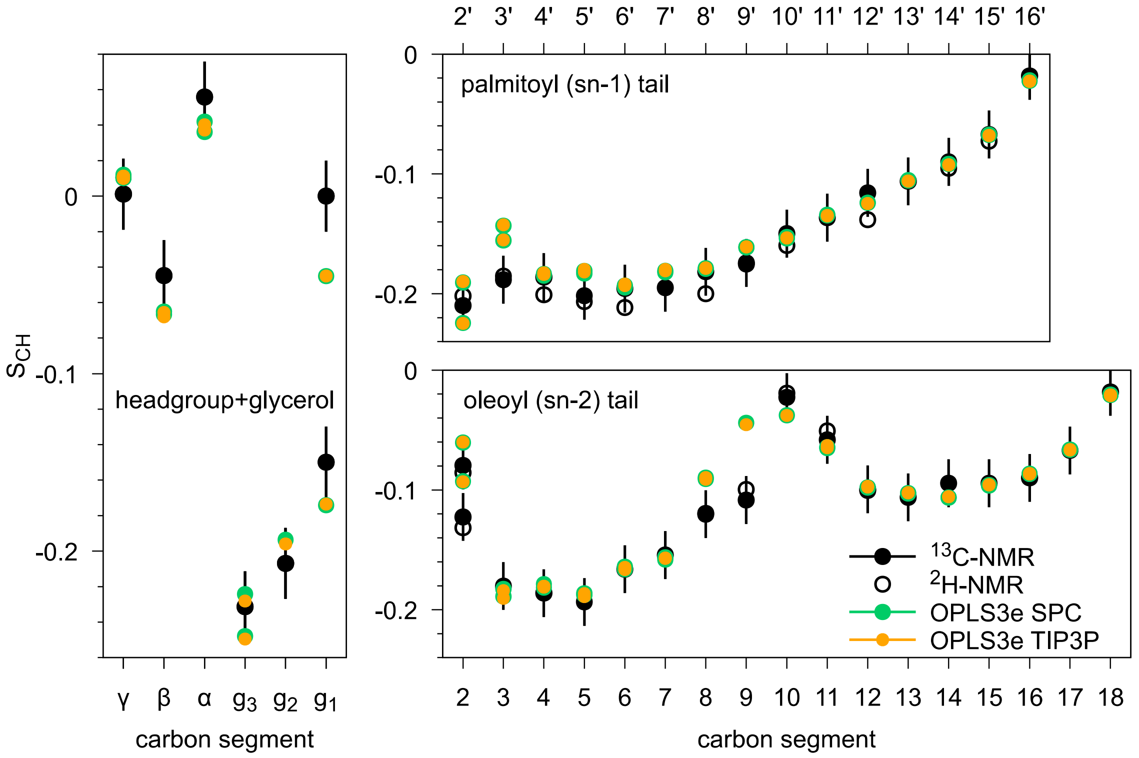

Figure S1. Order parameters at full hydration for headgroup, backbone, and acyl chains in OPLS3e with SPC and TIP3P water models. Experimental values for the POPC (^{1}H- ^{13}C NMR) at 300 K are from Ref. 1 and ^{2}H-NMR are from Ref. 2. Simulations are also for POPC.

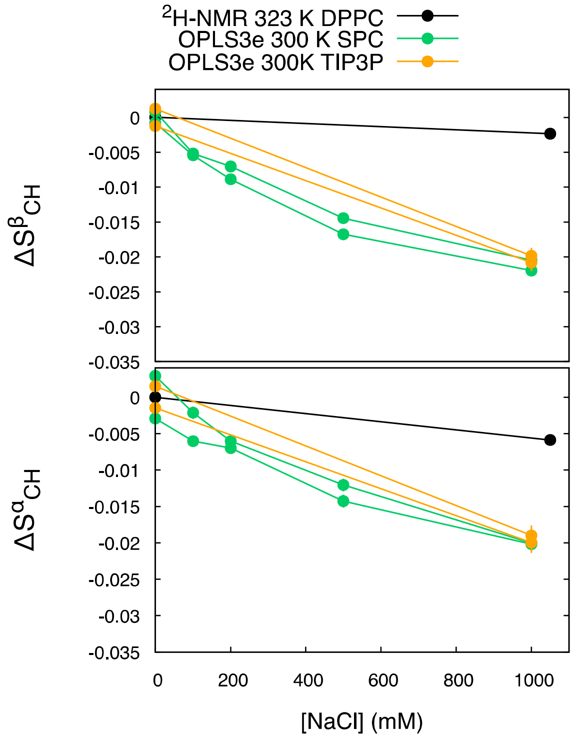

Figure S2. Change of order parameters in headgroup α and β segments in response to rising concentration of NaCl. Experimental values for DPPC (^{2}H NMR) at 323 K are from Ref. 3. Simulations POPC. The average C–H bond order parameters of R and S hydrogens were used to calculate the difference between the baseline and different salt concentrations.

2. Order parameters in response to additional salt

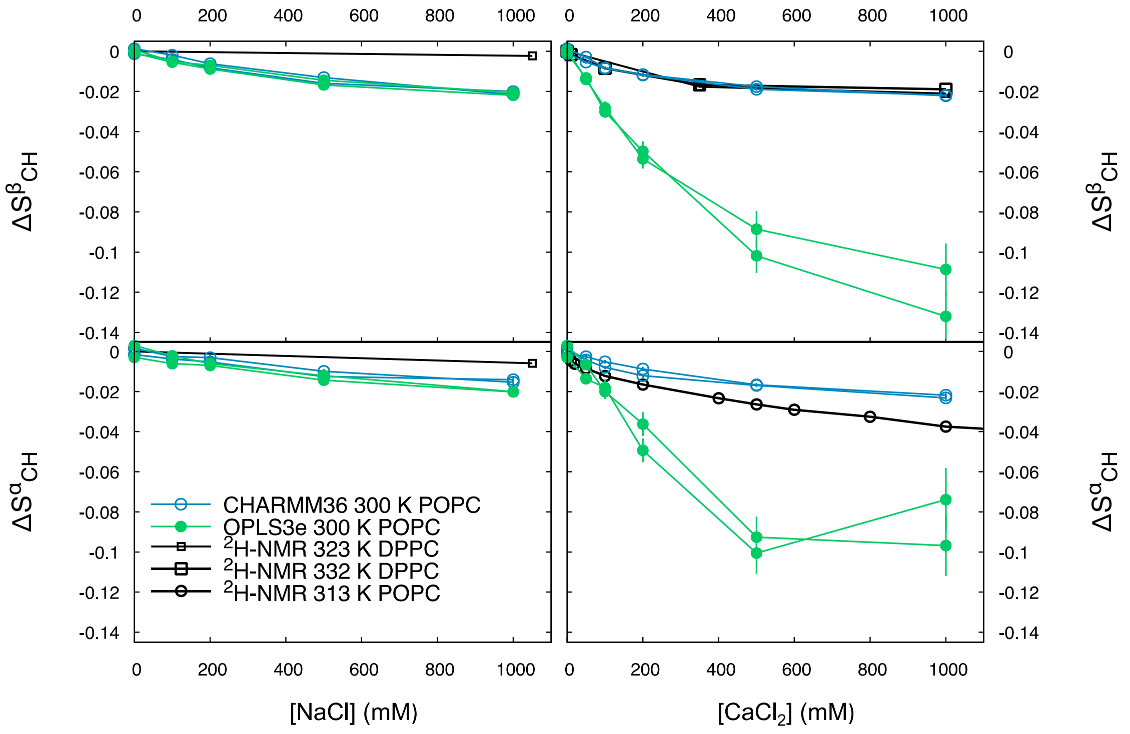

Figure S3. Change of order parameters in the headgroup α and β segments in response to rising concentration of NaCl or $CaCl_2$ as a full figure. Experimental values for DPPC (^{2}H NMR) at 323 K and 332 K are from Ref. 3 and for POPC (^{2}H NMR) at 313 K are from Ref. 4. The average C–H bond order parameters of R and S hydrogens were used to calculate the difference between the baseline and different salt concentrations.

3. Equilibration times of the ion binding and impact on order parameters

Our simulations show that quite long equilibration time is needed for ion binding to the membrane in OPLS3e (both Ca^{2+} and Cl^{-}) as the number of ions keeps rising in the vicinity of membrane (Fig. S4). 200 mM and higher concentrations seem to require (almost) the whole 1 μs simulation to reach final equilibrium. Catte et al. reported similar results over 1000 ns equilibration time for $CaCl_2$ in original CHARMM36 (no NBfix) (5). In our simulations, CHARMM36 with NBfix parameters does not induce ions to bind to membrane with high affinity and equilibration is consequently faster (Fig. S5). S^{α}_{CH} and S^{β}_{CH} also show decline as a function of simulation time in OPLS3e as the number of ions in vicinity of the membrane keeps rising, see Fig. 4 but not in CHARMM36 (Fig. S5).

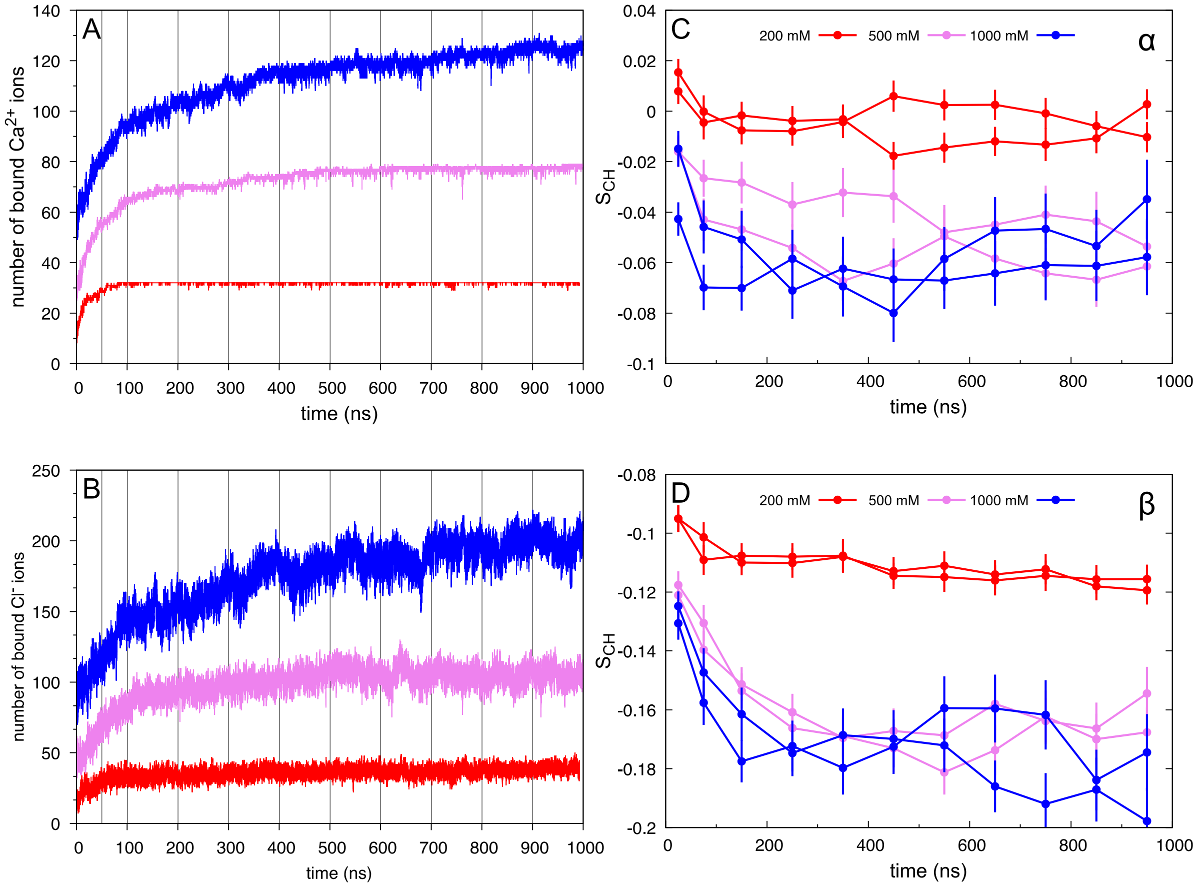

Figure S4. Number of (A) Ca^{2+} (B) Cl^{-} ions within 2.5 nm from bilayer center. (C) S^{α}_{CH} and (D) S^{β}_{CH} as a function of simulation time in OPLS3e with additional $CaCl_2$ 200, 500, and 1000 mM concentrations. Order parameters in C and D panels calculated for trajectory pieces shown with vertical lines in A and B panels.

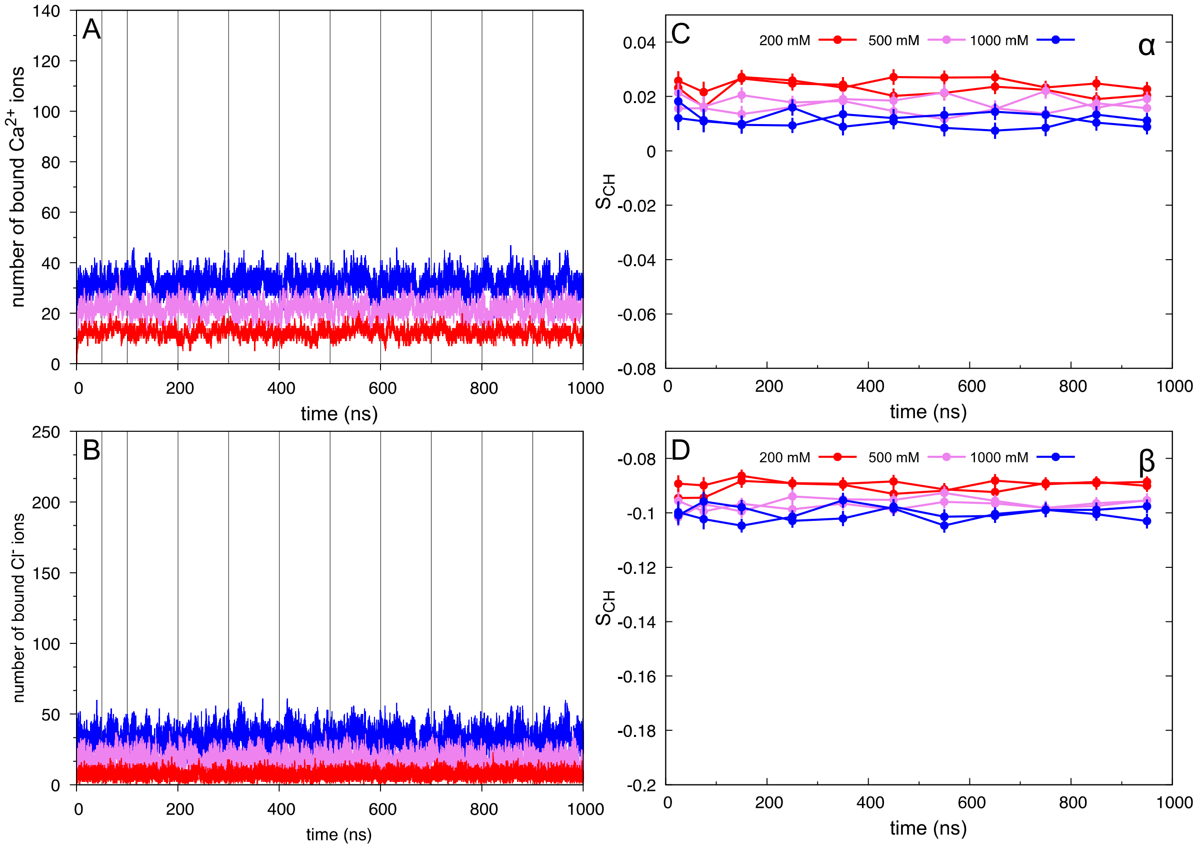

Figure S5. Number of (A) Ca^{2+} (B) Cl^- ions within 2.5 nm from bilayer center. (C) S^{α}_{CH} and (D) S^{β}_{CH} as a function of simulation time in CHRMM36 with additional $CaCl_2$ 200, 500, and 1000 mM concentrations. Order parameters in C and D panels calculated for trajectory pieces shown with vertical lines in A and B panels.

4. Scaling the ion charge in OPLS3e

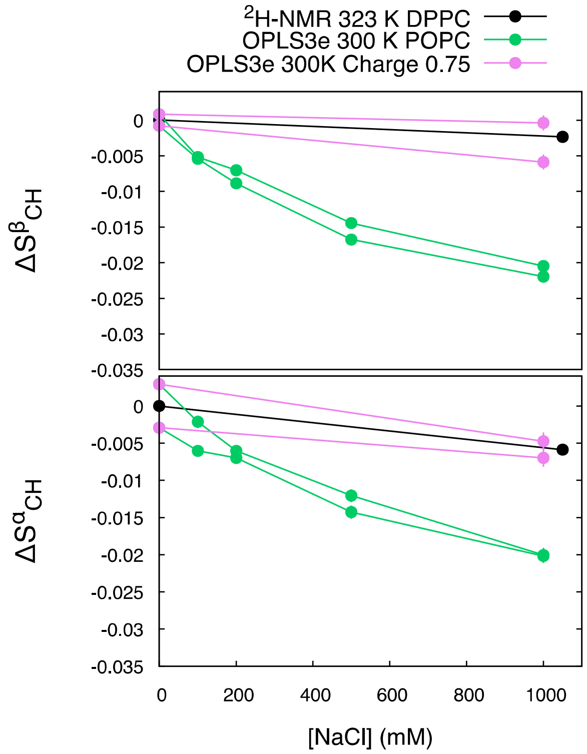

Figure S6. Change of order parameters in the POPC headgroup α and β segments in response to additional NaCl in OPLS3e force field with scaled and unscaled ions. Scaling was done with the factor of 0.75 for the charge of both Na^+ and Cl^- ions. Experimental values for DPPC (^{2}H NMR) at 323 K are from Ref. 3. The average C–H bond order parameters of R and S hydrogens were used to calculate the difference between the baseline and different salt concentrations.

Over-binding of ions to membranes is a problem in most of the current force fields and based on our studies this is the case also with OPLS3e force field. One reason behind the over binding might be too high charge of the ions. In previous studies, it has been proposed that scaling the ion charge might help to overcome mistakes resulting from the lacking electronic polarization (6, 7). In electronic continuum correlation (ECC) theory, based on quantum mechanical calculations, scaling factor of ions is 0.75. This scaling of ion charges has been able to improve monovalent ion binding in some force fields, but has not been sufficient for the divalent $CaCl_2$. To further investigate behavior of ions in OPLS3e, we scaled the charge of monovalent NaCl and divalent $CaCl_2$ by factor 0.75, as in ECC theory, and performed simulations using 1000 mM concentrations with scaled charges. Scaling enhanced order parameters for the alpha and beta carbons in the presence of additional 1000 mM NaCl (Fig. S6) but was not sufficient for $CaCl_2$ (Fig. S7). However, with scaled charged of $CaCl_2$, ion binding to membrane equilibrates much faster than with unscaled charges (Fig. S8).

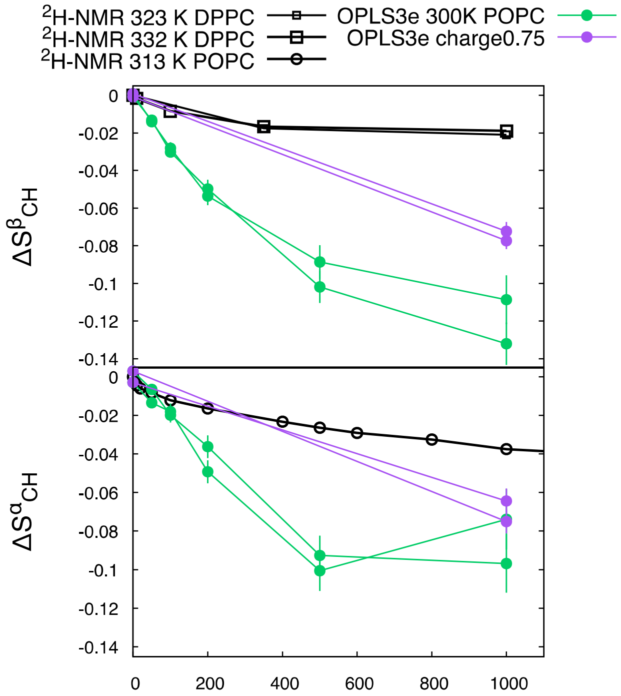

Figure S7. Change of order parameters in the POPC headgroup α and β segments in response to additional $CaCl_2$ in OPLS3e force field with scaled and unscaled ions. Scaling was done with the factor of 0.75 for the charge of both Ca^{2+} (charge 1.5) and Cl^- ions. Experimental values for DPPC (^{2}H NMR) at 323 K and 332 K are from Ref. (3) and for POPC (^{2}H NMR) at 313 K are from Ref. (4). The average C–H bond order parameters of R and S hydrogens were used to calculate the difference between the baseline and the different salt concentrations. Last 100 ns of 1000 ns trajectory was used for analysis for unscaled ions, and last 400 ns of 500 ns trajectory for scaled ions.

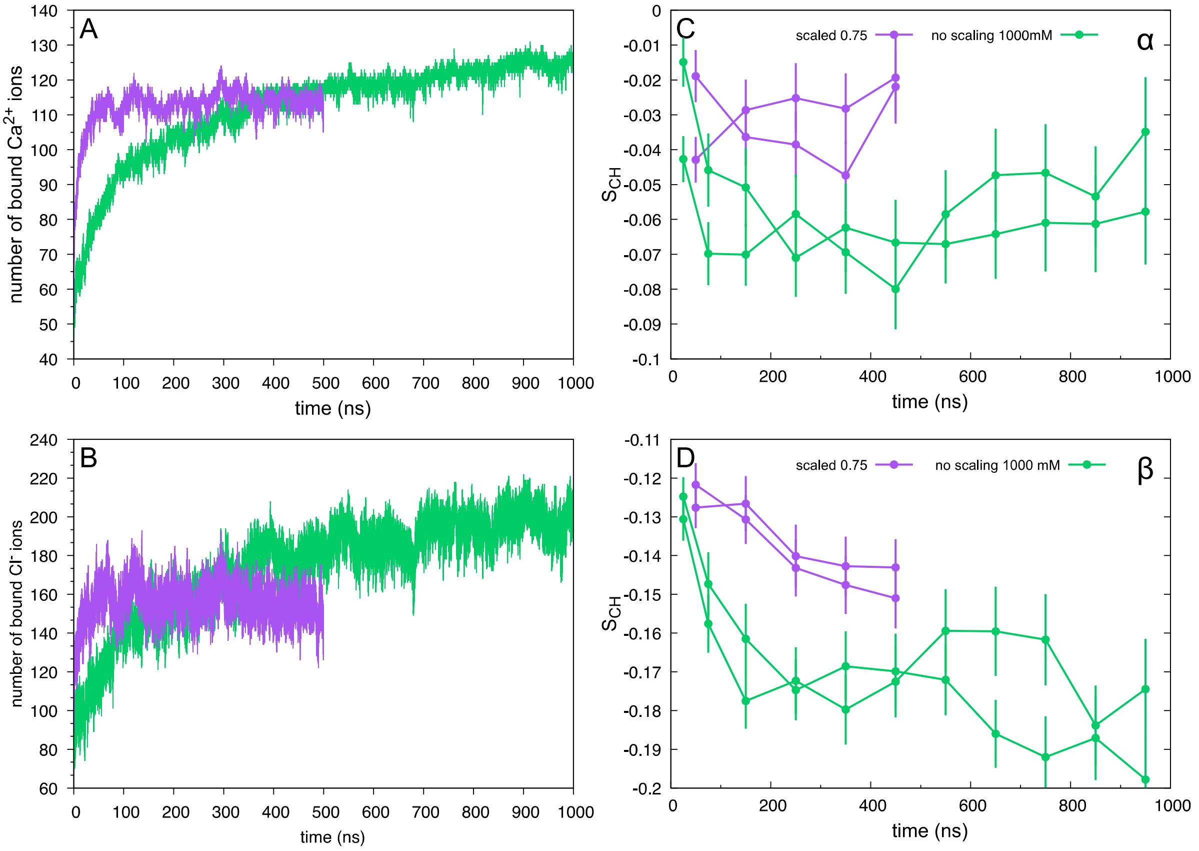

Figure S8. Number of (A) Ca^{2+} (B) Cl^{-} ions within 2.5 nm from bilayer center. (C) S^{α}_{CH} and (D) S^{β}_{CH} as a function of simulation time in OPLS3e with additional 1000 mM scaled and unscaled $CaCl_2$.

The over binding of Ca^{2+} cannot be easily fixed by scaling the charge of the ions (or by adding non-bonded fix parameters). To this end, Melcr et al. extended the ECC theory to include also partial charges of the polar region of POPC phospholipids to build the ECC-POPC model based on the Lipid14 force field (they also did this for CHARMM36 with same scaling) (7). The resulting ECC-POPC model produces experimental order parameter responses to NaCl and even for divalent $CaCl_2$ unlike any other current force field, and can thus be considered as one of the most realistic models to describe POPC lipid membrane in the presence of ions so far.

5. Small bug in CHARMM36 parameters

We noticed a small bug in the CHARMM36 force field parameters obtained at the time of the research (summer 2020) from CHARMM-GUI. Parameters were missing following dihedral line:

```
316a317
> CTL2  CEL1  CEL1  HEL1   9 1.800000e+02 2.510400e+01   2
```

By the end of 2020, the parameters in CHARMM-GUI were fixed. We compared the order parameters from simulations performed with parameters without (bug) and with the dihedral (bugfix) and discovered that there was no difference in order parameters between those parameter sets (Fig S9). However, we have used CHARMM36 bugfix parameters in all the simulations presented in this paper.

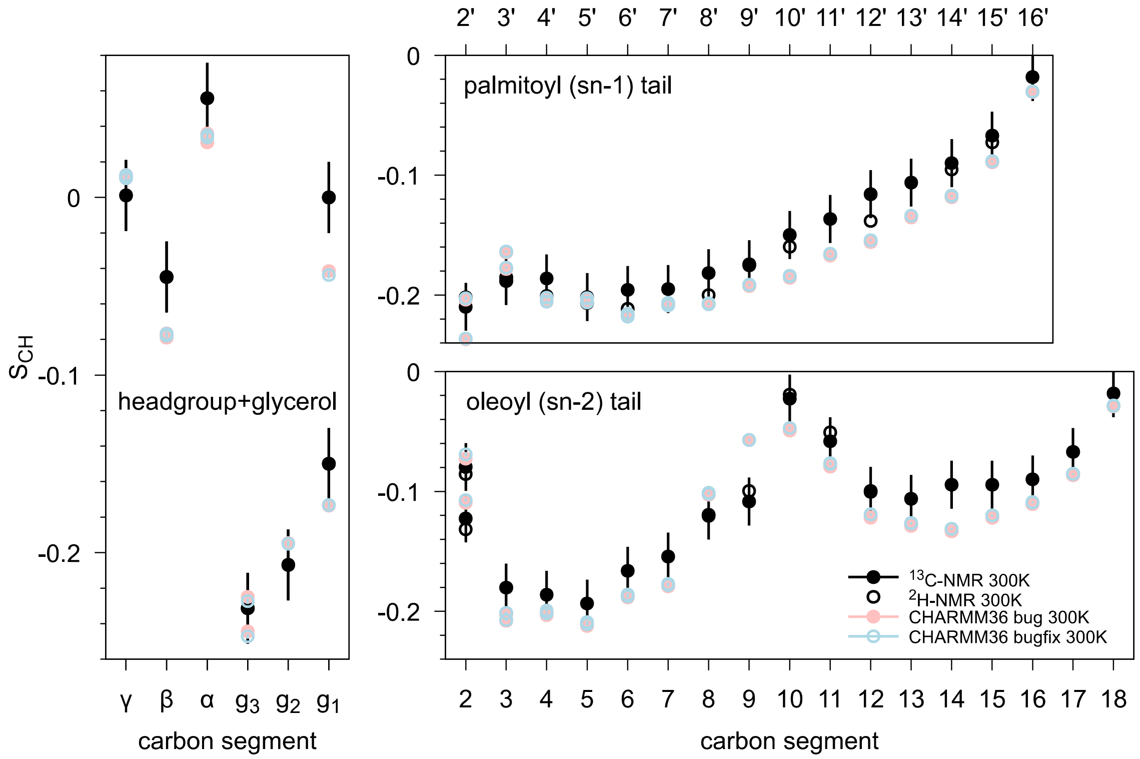

Figure S9. Order parameters for POPC in CHARMM36 with a bug compared to parameters with bugfix for headgroup, backbone, and acyl chains. Experimental values for the POPC (^{1}H-^{13}C NMR) at 300 K are from Ref. 1 and ^{2}H-NMR are from Ref. 2.

6. Effect of temperature change in CHARMM36

In addition to 300 K, we simulated some CHARMM36 systems also using 314 K temperature. We can see that order parameters at full hydration are in general slightly higher at 314 K than 300 K (Fig. S10). Lowering hydration induces similar responses in both temperatures, but 314 K does not induce forking in 5 w/l hydration in contrast to 300 K (Fig. S11). Slight changes in order parameters might be linked to temperature-induced changes in area per lipid. At 314 K the area per lipid is larger, which allows lipids to move more than at 300 K (Fig. S12).

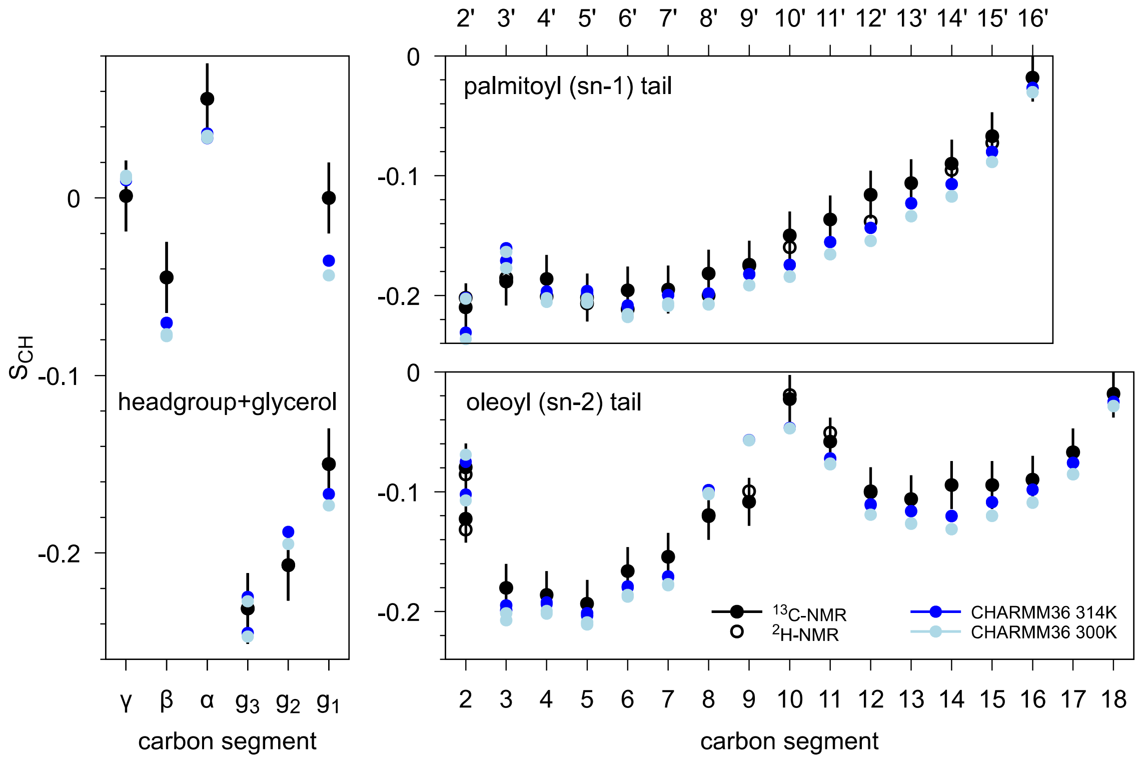

Figure S10. Order parameters at full hydration (33 waters/lipid) for POPC headgroup and backbone acyl chains in simulations and experiments. Experimental values for the POPC (^{1}H-^{13}C NMR) at 300 K are from Ref. 1 and ^{2}H-NMR are from Ref. 2.

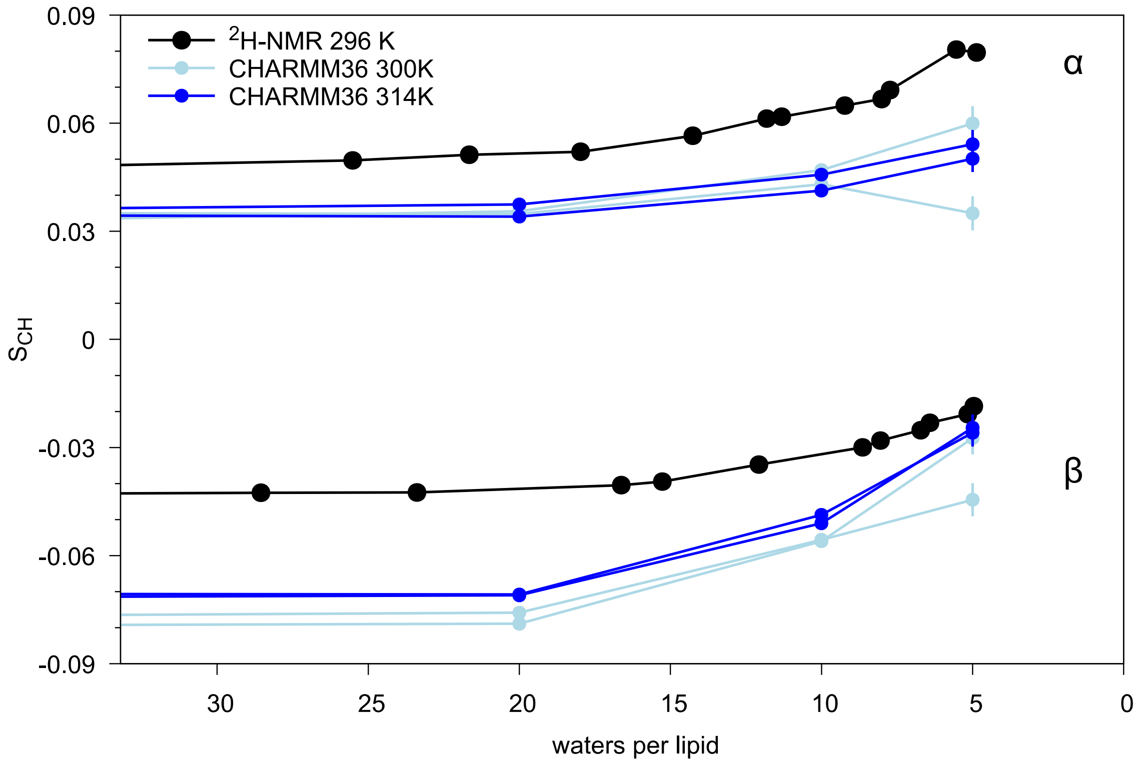

Figure S11. Order parameters in response to decreasing hydration level for the headgroup α and β carbons. Experimental values for POPC (^{2}H NMR) at 296 K are from Ref. (8).

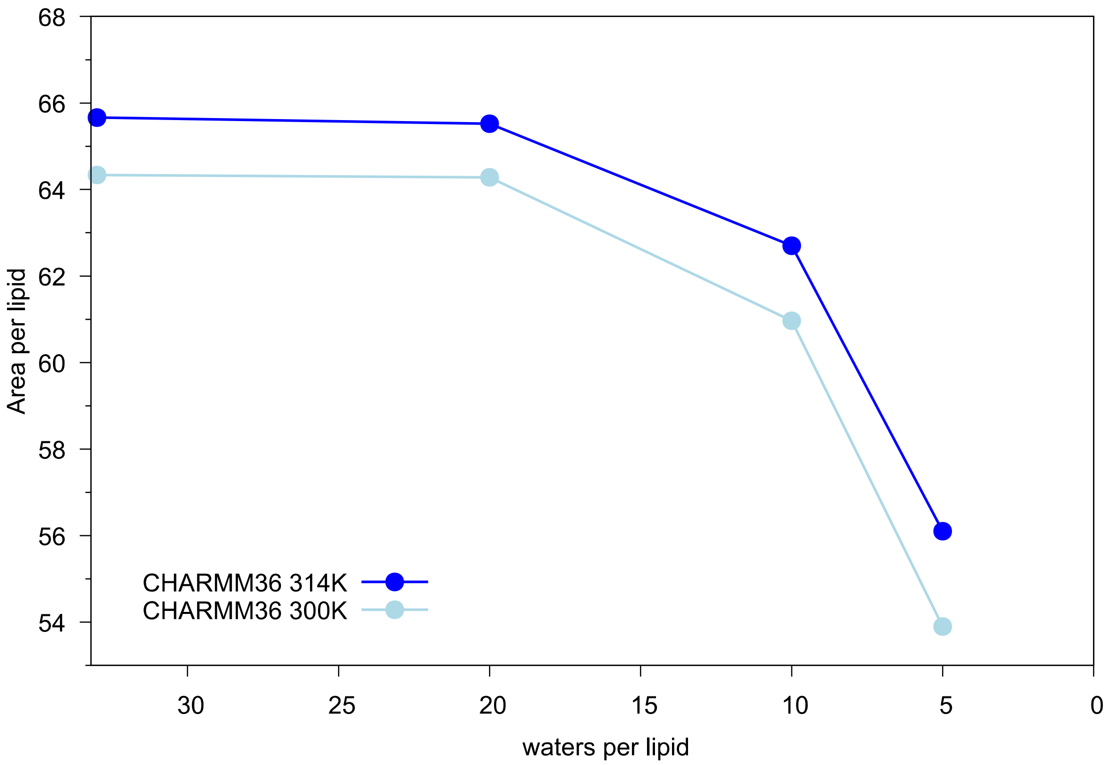

Figure S12. Area per lipid in response to lowering hydration level in CHARMM36 with 300 K and 314 K temperatures.

7. Area per lipid as a function of time

Area per lipid was used in this study to determine when the simulation systems had reached equilibrium. Other structural properties in addition to area per lipid (e.g., order parameters and density distributions) do not usually change significantly after area per lipid reaches a stable value (9). For simulations with ions we also waited for the ion distribution between the solvent and the membrane to stabilize which generally took longer than the stabilization of the area per lipid. In the case of $CaCl_2$, we could not reach the equilibrium in the ion distribution in the 1000 ns production run. In most simulations area per lipid reaches a stable value during first 100 ns of the simulation (Fig S13.) Exceptions are systems with low hydration (5 w/l), and OPLS3e with additional $CaCl_2$ for which area per lipid equilibrates significantly slower.

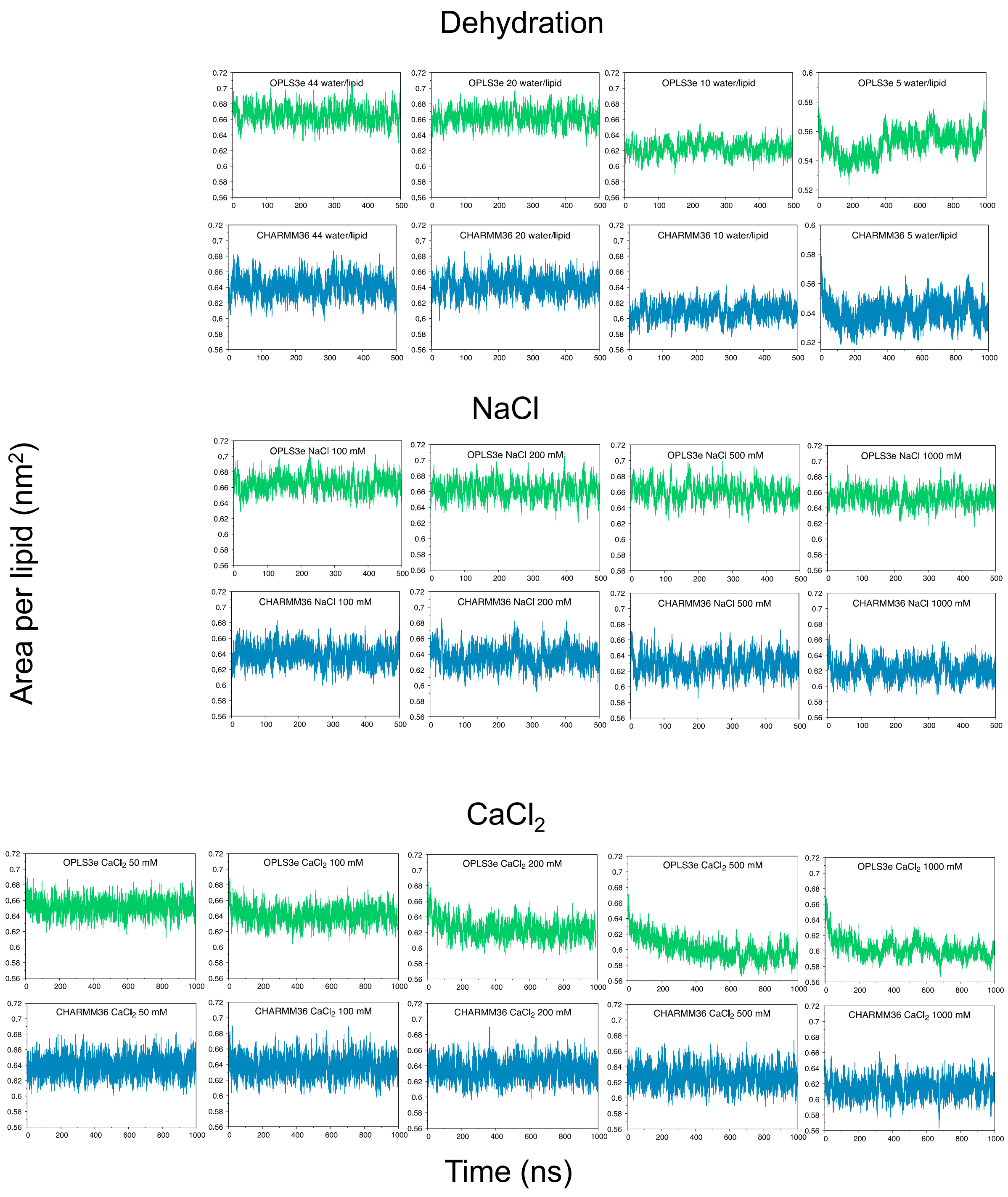

Figure S13. Area per lipid for each simulation as a function of time.

8. Lipid mean squared displacement (MSD)

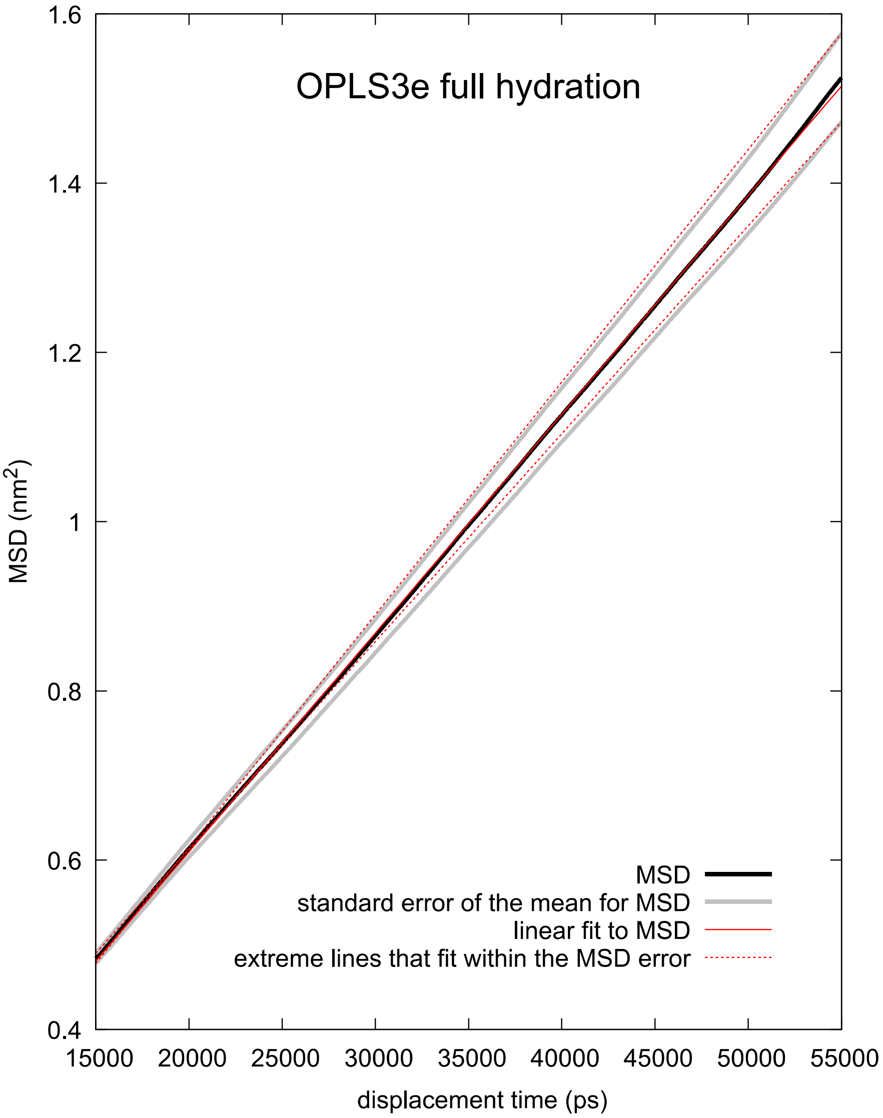

Figure S14. Lateral mean squared displacement (MSD, black line) of the center of mass for POPC lipids at full hydration simulated using the OPLS3e force field. The displacement time range shown indicates the visually linear region, over which a linear fit of the MSD (red) was performed. The gray lines show the standard error of the mean (SEoM) for MSD, and the dotted red lines are the lines with extremal slopes that could fit within the SEoM bounds, and thus provide natural error estimates for the MSD slope.

References

1. Ferreira TM, Coreta-Gomes F, Ollila OHS, Moreno MJ, Vaz WLC, Topgaard D. Cholesterol and POPC Segmental Order Parameters In Lipid Membranes: Solid State ^{1}H–^{13}C NMR And MD Simulation Studies. Phys Chem Chem Phys. 2013;15(6):1976-89.

2. Seelig J, Waespe-Sarcevic N. Molecular Order in Cis And Trans Unsaturated Phospholipid Bilayers. Biochemistry. 1978;17(16):3310-5.

3. Akutsu H, Seelig J. Interaction of Metal Ions With Phosphatidylcholine Bilayer Membranes. Biochemistry. 1981;20(26):7366-73.

4. Altenbach C, Seelig J. Calcium Binding to Phosphatidylcholine Bilayers As Studied By Deuterium Magnetic Resonance. Evidence For the Formation Of A Calcium Complex With Two Phospholipid Molecules. Biochemistry. 1984;23(17):3913-20.

5. Catte A, Girych M, Javanainen M, Loison C, Melcr J, Miettinen MS, et al. Molecular Electrometer and Binding of Cations to Phospholipid Bilayers. Phys. Chem. Chem. Phys. 2016;18(47):32560-9.

6. Kohagen M, Mason PE, Jungwirth P. Accounting for Electronic Polarization Effects in Aqueous Sodium Chloride via Molecular Dynamics Aided by Neutron Scattering. J. Phys. Chem. B. 2016;120(8):1454-60.

7. Melcr J, Martinez-Seara H, Nencini R, Kolafa J, Jungwirth P, Ollila OHS. Accurate Binding of Sodium and Calcium to a POPC Bilayer by Effective Inclusion of Electronic Polarization. J. Phys. Chem. B. 2018;122(16):4546-57.

8. Bechinger B, Seelig J. Conformational Changes of The Phosphatidylcholine Headgroup Due To Membrane Dehydration. A ^{2}H-NMR Study. Chemistry and Physics of Lipids. 1991;58(1):1-5.

9. Lyubartsev AP, Rabinovich AL. Force Field Development for Lipid Membrane Simulations. Biochim. Biophys. Acta Biomembr.. 2016;1858(10):2483-97.
